# An Anterior Cingulate Cortex‐Anterior Insular Cortex Glutamatergic Circuit Gates Stress‐Induced Visceral Hypersensitivity and Anxiety via Ionotropic Glutamate Receptors Trafficking

**DOI:** 10.1002/advs.76298

**Published:** 2026-07-29

**Authors:** Junwen Wang, Guangbing Duan, Huihui Sun, Zhiyu Dong, Qiwei Wang, Xiaowei Li, Ying Chen, Shuchang Xu, Ying Huang

**Affiliations:** ^1^ Department of Gastroenterology Tongji Institute of Digestive Diseases Tongji Hospital School of Medicine Tongji University Shanghai China; ^2^ Fundamental Research Center Shanghai YangZhi Rehabilitation Hospital (Shanghai Sunshine Rehabilitation Center), School of Medicine Tongji University Shanghai China; ^3^ Department of Gastrointestinal Endoscopy Huadong Hospital Fudan University Shanghai China; ^4^ Department of Gastrointestinal Surgery Shanghai Baoshan Hospital of Integrated Traditional Chinese and Western Medicine Shanghai University of Traditional Chinese Medicine Shanghai China

**Keywords:** anterior cingulate cortex, anterior insular cortex, anxiety, glutamatergic neuronal circuit, visceral hypersensitivity

## Abstract

The comorbidity between irritable bowel syndrome (IBS) and anxiety arises from impaired sensory‐emotional integration, yet its underlying neural circuit mechanisms remain elusive. Using a water‐avoidance stress (WAS) rat model, this study identifies a glutamatergic projection from the anterior cingulate cortex (ACC^Glu^) to the anterior insular cortex (AIC^Glu^) that mediates stress‐induced visceral hypersensitivity and anxiety. Chemogenetic or optogenetic manipulation reveals that activation of glutamatergic neurons in AIC or the ACC^Glu^‐AIC^Glu^ circuit mimics WAS‐induced both visceral hypersensitivity and anxiety. Conversely, inhibition of this circuit reverses WAS‐induced visceral hypersensitivity and anxiety. However, bidirectional chemogenetic manipulation of ACC^Glu^‐AIC^Glu^ circuit fails to affect ovalbumin‐induced visceral hypersensitivity, indicating stress‐specific modulation. Additionally, at synaptic level, WAS rats exhibit elevated synaptosomal expression of GluA1/A3‐containing AMPARs and NR2B‐containing NMDARs of ionotropic glutamate receptors (iGluRs) in the AIC, accompanied by enhanced AMPAR‐ and NMDAR‐mediated currents. These alterations are alleviated by chemogenetic inhibition of the ACC^Glu^‐AIC^Glu^ pathway. Moreover, inhibition of GluA1/A3 and NR2B receptors relieved visceral hypersensitivity and anxiety in WAS rats. Taken together, these findings reveal that AMPA and NMDA receptor trafficking driven by this circuit underlies stress‐induced comorbidity of visceral pain and anxiety, uncovering a circuit‐to‐synapse mechanism and highlighting potential therapeutic targets for circuit‐based treatment of this comorbidity.

## Graphical abstract

This study identifies an anterior cingulate cortex (ACC) to anterior insular cortex (AIC) glutamatergic (Glu) circuit that gates stress induced visceral hypersensitivity and anxiety. Circuit activation induces both phenotypes, whereas inhibition relieves them. Mechanistically, this circuit acts via trafficking of ionotropic glutamate receptors (AMPAR and NMDAR) at synapses, uncovering a shared substrate for stress related visceral hypersensitivity and anxiety.

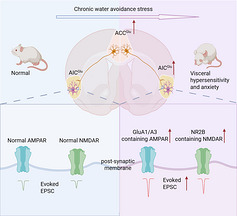

## 1 Introduction

The neural mechanisms underlying the complex interplay between sensation and emotion in the brain represent a fundamental question in systems neuroscience. This sensory‐emotional integration is particularly salient in pain, which is inherently both a sensory and an emotional experience [1, 2]. The high prevalence of pain‐psychiatric comorbidity represents a major challenge in both clinical and basic research, underscoring the existence of shared pathophysiology mechanisms [3].

Irritable bowel syndrome (IBS), a typical disorder of gut‐brain interaction characterized by chronic abdominal pain accompanied by alterations in bowel habits, provides an excellent model to investigating this sensory‐emotional interaction [4]. Visceral hypersensitivity, defined as enhanced sensory responsiveness to intra‐abdominal stimuli, has important contributions to the development of IBS‐related symptoms, including chronic colorectal pain [5]. In most clinical cases, IBS patients report the presence of psychosocial comorbidities, which diminish the therapeutic efficacy of analgesic treatment and impair patients’ social functioning. Anxiety disorders have been identified as a prevalent psychiatric comorbidity in IBS patients, with the pooled prevalence rate of anxiety symptoms in those patients estimated at 39.1% [6]. A recent Genome‐Wide Association study has revealed congruent genetic vulnerabilities between IBS and anxiety disorders [7]. Longitudinal studies have established a correlation between anxiety disorders and the development of IBS [6, 8, 9]. Anxiolytics such as tandospirone, as well as psychotherapies including cognitive‐behavioral therapy, have been shown to ameliorate IBS symptoms [10, 11]. Notably, alterations in supraspinal structures, especially the abnormal firing of neurons of the brain regions, as well as the inter‐regional communication within these brain regions, have been proposed as shared pathophysiological mechanisms underlying anxiety and visceral hypersensitivity [12]. These findings suggest that IBS and anxiety may have cross‐talk in pathogenic mechanisms among these brain regions. A critical unresolved question, therefore, is to elucidate the neural circuit mechanisms by which a single pathological process coordinately generates both visceral pain and anxiety.

The cortical brain regions are the ultimate hubs for pain sensation, with the anterior cingulate cortex (ACC) being a region of significant interest in the studies of chronic pain [13]. Previous animal studies have shown that electrophysiological and molecular alterations in ACC contribute to the pathogenesis of both visceral hypersensitivity as well as anxiety [14, 15, 16, 17]. ACC acts as a central hub in both pain perception and emotional regulation due to its extensive connections with a variety of cortical regions, including prefrontal, insular, and visual cortex, and subcortical regions such as the thalamus, amygdala, periaqueductal gray, and claustrum [13, 18]. However, it is unclear the precise role of the ACC‐related circuit in comorbid visceral hypersensitivity and anxiety.

Functional magnetic resonance imaging (fMRI) studies have identified distinct alterations within several brain networks in IBS patients, especially in the salience network (SN). These alterations are proposed to be associated with the biases of threat assessment of stimuli and consequence anticipation [19, 20, 21, 22]. The ACC and the anterior insular cortex (AIC), have been characterized as pivotal nodes of the SN [23, 24]. It has been shown that an increase in rectal stimulation pressure results in enhanced activation within the SN, specifically in the ACC and AIC. Comparative analyses have revealed that IBS patients exhibit augmented activity in these regions compared with healthy controls [21, 22]. Additionally, it has been reported that the intrinsic connectivity of SN alters in individuals with comorbid anxiety disorder [25, 26]. While anatomical tracing studies in rodent models have provided evidence for the anatomical connection between the ACC and AIC, it remains unclear for the specific types of projection neurons and their functional roles in this circuit [27, 28, 29, 30].

In this study, using chemogenetic and optogenetic neuronal manipulation combined with in vitro electrophysiological recordings and behavior tests, we aimed to determine the specific role of ACC, AIC, and ACC‐AIC circuit in water avoidance stress (WAS)‐induced comorbidity of visceral hypersensitibity and anxiety. Furthermore, we assessed the alterations of ionotropic glutamate receptors (iGluRs) in AIC induced by WAS, and investigated whether the ACC‐AIC circuit is involved in the process.

## 2 Results

### 2.1 WAS Produces Anxiety‐Like Behavior and Visceral Hypersensitivity but not Depression‐Like Behavior

**FIGURE 1:**
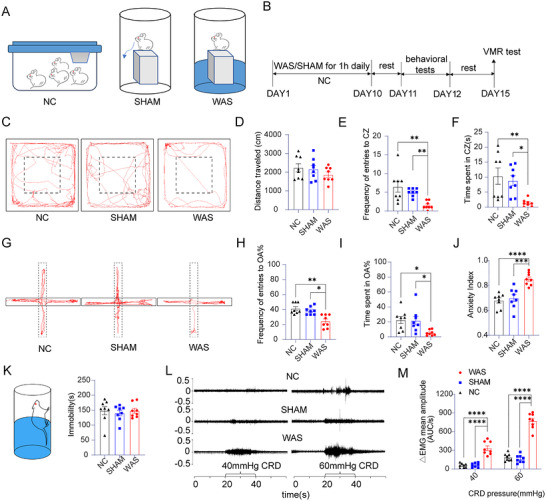
WAS induces anxiety‐like behavior and visceral hypersensitivity, but not depression‐like behavior. (A) WAS model paradigm. NC: normal control, SHAM: sham water‐avoidance stress, WAS: water‐avoidance stress. (B) Timeline of WAS model induction and behavioral tests. VMR: visceral motor response. (C–F) Results of the open‐field test (OFT). Representative exploration traces in (C), analysis of total distance in (D), analysis of frequency of entries into the center zone (CZ) in (E), analysis of time spent in the CZ in (F). (one‐way ANOVA with Tukey's post‐hoc test, D, F _(2, 21)_ = 1.017, *p* = 0.3789; Kruskal–Wallis test with Dunn's post‐hoc test, E, *p* = 0.0012; F, *p* = 0.0017.) (G–J) Results of elevated plus maze (EPM).Typical exploration traces in (G), analysis of percentage of entries into open arms (OA) in (H), analysis of percentage of time spent in the OA in (I), statistical results of the Anxiety index calculated based on EPM behavioral parameters in (J). Anxiety index = 1‐ [(time spent in OA/total time on maze) + (number of entries to the OA/total exploration on maze)/2]. (Kruskal–Wallis test with Dunn's post‐hoc test, H, *p* = 0.0012; one‐way ANOVA with Tukey's post‐hoc test, I, F _(2, 21)_ = 4.949, *p* = 0.0173; J, F _(2, 21)_ = 16.54, *p* < 0.0001.) (k) Forced‐swimming test (FST) paradigm(left) and the statistical results of immobility time during the last 4 min in FST (right). (one‐way ANOVA, F _(2, 21)_ = 0.1742, *p* = 0.8413.) (L–M) VMR results to 40mmHg and 60mmHg CRD stimulation. CRD: colorectal distention. Representative external abdominal oblique muscle EMG recordings in (L), analysis of the mean amplitude of △EMG in (M) (one‐way ANOVA with Tukey's post‐hoc test, M, 40mmHg, F _(2, 21)_ = 44.30, *p* < 0.0001; 60mmHg, F _(2, 21)_ = 120.3, *p* < 0.0001). Data presented as mean ± SEM (*n* = 8 rats). *p < 0.05, **p < 0.01, ***p < 0.001, ****p < 0.0001.

Chronic WAS was used to establish a model of comorbid anxiety and visceral hypersensitivity (Figure 1A). Following exposure to WAS for ten days, the rats were assessed anxiety‐like behavior by open‐field test (OFT) and elevated plus maze (EPM) test (Figure 1B). In the OFT, compared with the sham‐WAS (SHAM) and normal control (NC) groups, WAS rats displayed fewer entries to center zone (CZ) and spent less time in CZ without the decrease of total travel distance (Figure 1C–F). Consistent with the OFT results, WAS rats showed shorter time and fewer number of entries in the EPM open arms (OA) (Figure 1G–I). Meanwhile, the anxiety index was significantly increased in WAS rats compared with that of SHAM and NC rats (Figure 1J). However, there was no difference in anxiety‐like behavior between the SHAM and NC groups (Figure 1C–J).

The depression‐like behavior was then tested using forced swimming test (FST). The immobility time in the FST did not change in WAS and SHAM rats, suggesting that there was no difference of depression in the three groups of rats (Figure 1K).

Finally, the visceral motor response (VMR) tests showed that visceral sensation to both 40 and 60 mmHg colorectal distention (CRD) pressures were enhanced in WAS rats compared with that of SHAM and NC rats (Figure 1L,M). Whereas no significant difference in visceral sensation between the SHAM and NC groups was found (Figure 1L,M).

Together, these findings suggest that WAS rats developed anxiety‐like behavior and visceral hypersensitivity but not depression‐like behavior.

### 2.2 Activation of ACC Glutamatergic Neurons (ACC^Glu^) is Crucial in WAS‐Induced Visceral Hypersensitivity and Anxiety‐Like Behavior

**FIGURE 2:**
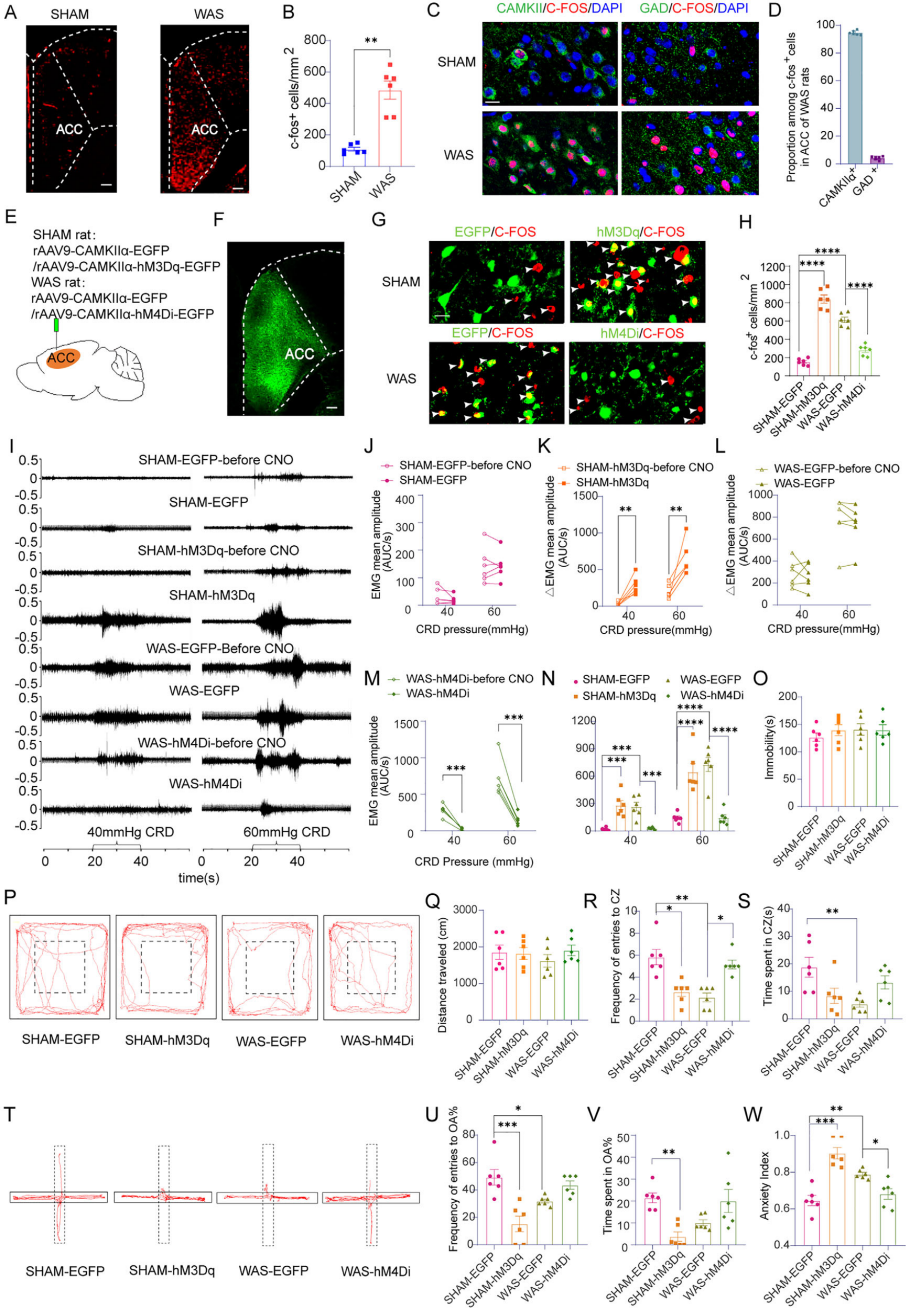
The activation of ACC^Glu^ is crucial in WAS‐induced visceral hypersensitivity and anxiety‐like behaviors. (A,B) C‐fos expression in the ACC area in WAS and SHAM rats. Representative images of colocalization in (A), statistical results in (B), Scale bar, 500 µm (unpaired Student's *t‐*test, B, t _(5.439)_ = 6.391, *p* = 0.0010). (C,D) The colocalization of c‐fos positive neurons with the glutamatergic neuronal marker CAMKIIα or the GABAergic neuronal marker GAD65+67 in the ACC of WAS and SHAM rats (red: c‐fos, blue: DAPI, green: CAMKIIα (left)/GAD65+67(right)).Representative images of colocalization in (C), statistical results in (D), Scale bar, 20 µm.(E) Schematic of bilateral ACC injection of rAAV virus injections into WAS or SHAM rats. (F) Typical image of ACC‐injection sites. Scale bar, 500 µm. (G,H) C‐fos expression in ACC neurons post‐virus expression. Representative images of colocalization in (G), Statistical results in (H), Scale bar, 20 µm. (one‐way ANOVA with Tukey's post‐hoc test, H, F _(3, 20)_ = 106.7, *p* < 0.0001). (I–N) Activation of ACC^Glu^ induced visceral hypersensitivity, while suppression of ACC^Glu^ alleviated WAS‐induced visceral hypersensitivity. Representative external abdominal oblique muscle EMG recordings from SHAM‐EGFP, SHAM‐hM3Dq, WAS‐EGFP and WAS‐hM4Di rats before and after intraperitoneal CNO injection in (I), analysis of the mean amplitude of △EMG in (J–N) (paired Student's *t*‐test, J, 40mmHg, t _(5)_ = 1.304, *p* = 0.2490; 60mmHg, t _(5)_ = 0.0056, *p* = 0.9957; K, 40mmHg, t _(5)_ = 4.667, *p* = 0.0055; 60mmHg, t _(5)_ = 4.452, *p* = 0.0067; L, 40mmHg, t _(5)_ = 0.3586, *p* = 0.7345; 60mmHg, t _(5)_ = 1.483, *p* = 0.1982; M, 40mmHg, t _(5)_ = 9.175, *p* = 0.0003; 60mmHg, t _(5)_ = 8.515, *p* = 0.0004; one‐way ANOVA with Tukey's post‐hoc test, N, 40mmHg, F _(3, 20)_ = 17.05, *p* < 0.0001; 60mmHg, F _(3, 20)_ = 25.21, *p* < 0.0001). (O) Immobility time during FST (one‐way ANOVA with Tukey's post‐hoc test, F _(3, 20)_ = 0.5020, *p* = 0.6852). (P–S) Results of OFT. Representative exploration traces in (P), statistical results of total distance in (Q), analysis of frequency of entries to CZ in (R), analysis of time spent in CZ in (S) (one‐way ANOVA with Tukey's post‐hoc test, Q, F _(3, 20)_ = 0.5508, *p* = 0.6534; S, F _(3, 20)_ = 4.940, *p* = 0.01; Kruskal–Wallis test with Dunn's post‐hoc test, R, *p* = 0.0005). (T–W) Results of EPM. Representative exploration traces in (T), analysis of percentage of entries to OA in (U), analysis of percentage of time spent in the OA in (V), statistical results of the Anxiety index in (W) (one‐way ANOVA with Tukey's post‐hoc test, U, F _(3, 20)_ = 11.84, *p* = 0.0001; W, F _(3, 20)_ = 20.23, *p* < 0.0001; Kruskal–Wallis test with Dunn's post‐hoc test, V, *p* = 0.0030). Data presented as mean ± SEM (*n* = 6 rats). *p < 0.05, **p < 0.01, ***p < 0.001, ****p < 0.0001.

ACC is a region contributing to both pain perception and emotional regulation [31]. To examine whether the ACC was activated in the rats with visceral hypersensitivity and anxiety, we tested the expression of c‐fos in brain sections of the ACC region from WAS and SHAM rats. WAS rats showed more c‐fos‐positive cells in the ACC area, indicating that more ACC neurons were activated (Figure 2A,B). Additionally, we observed the colocalization of Calcium/Calmodulin‐dependent protein kinase II alpha (CAMKIIα, a specific marker for glutamatergic neurons) or Glutamate Decarboxylase 65+67 (GAD65+67, a specific marker for GABAergic neurons) with c‐fos to identify specific cell types of the activated neurons. The c‐fos‐positive cells in WAS rats were found to be CAMKIIα‐positive neurons rather than GAD‐positive GABAergic interneurons (Figure 2C,D).

To investigate the role of activated glutamatergic neurons in WAS‐induced visceral hypersensitivity and anxiety‐like behavior, we used the designer receptors exclusively activated by designer drugs (DREADD)‐based chemogenetics with the human M3 muscarinic acetylcholine receptor DREADD q mutant (hM3Dq) or the human M4 muscarinic acetylcholine receptor DREADD inhibitory mutant (hM4Di). The recombinant adeno‐associated virus (rAAV), rAAV9‐CAMKIIα‐hM3Dq‐enhanced green fluorescent protein (EGFP) or rAAV9‐CAMKIIα‐hM4Di‐EGFP virus, was injected into the bilateral ACC of SHAM (SHAM‐hM3Dq group) or WAS (WAS‐hM4Di group) rats, respectively. When hM3Dq or hM4Di was activated by Clozapine N‐oxide (CNO), CAMKIIα‐positive neurons would be activated or inhibited. As a control, the rAAV9‐CAMKIIα‐EGFP was bilaterally injected into the ACC of both SHAM (SHAM‐EGFP group) and WAS rats (WAS‐EGFP group) (Figure 2E). The injection sites and expression of viruses were validated post hoc via brain slices (Figure 2F). Chemogenetic effects were confirmed by the c‐fos expression after intraperitoneal injection of CNO. In SHAM groups, c‐fos expression increased greatly in the ACC of rats that had hM3Dq injection than those had EGFP injection. Additionally, the majority of hM3Dq‐expressing cells were co‐labeled with c‐fos (Figure 2G,H). Whereas in WAS groups, c‐fos expression decreased greatly in the ACC of rats that had hM4Di injection than those had EGFP injection. Moreover, the cells expressing hM4Di were rarely co‐labeled with c‐fos (Figure 2G,H). These results suggest the activation or suppression of ACC^Glu^ by hM3Dq or hM4Di, respectively.

The VMRs to CRD of each group were recorded before and after CNO injection (Figure 2I). In the SHAM‐EGFP and WAS‐EGFP groups, the VMRs to both 40 and 60mmHg CRD did not change regardless of CNO injection (Figure 2J,L). However, the VMRs of SHAM‐hM3Dq rats increased significantly (Figure 2K) whereas the VMRs of WAS‐hM4Di rats reduced (Figure 2M) after CNO injection compared with the baseline. Further comparison showed that after CNO injection, the VMRs of SHAM‐hM3Dq rats were higher than that of the SHAM‐EGFP rats, while the VMRs of WAS‐hM4Di rats were lower than that of the WAS‐EGFP rats (Figure 2N).

Anxio‐depression‐like behaviors of each group were also tested after CNO infusion. No significant differences were found in immobility time in FST (Figure 2O) and total travel distance in OFT (Figure 2P,Q), indicating the alteration of ACC activity did not affect depression or locomotion. However, the SHAM‐hM3Dq group displayed fewer entries to CZ compared with the SHAM‐EGFP group. The WAS‐hM4Di displayed more entries to CZ compared with the WAS‐EGFP group in OFT (Figure 2R). Although SHAM‐EGFP rats spent more time in the CZ than WAS‐EGFP rats, no significant differences were found in the time spent in the CZ between SHAM‐hM3Dq and SHAM‐EGFP or between WAS‐hM4Di and WAS‐EGFP (Figure 2S). For EPM, the SHAM‐hM3Dq rats entered into OA less frequently and spent less time in OA, whereas no significant difference in open arm exploration was found between WAS‐hM4Di and WAS‐EGFP rats. (Figure 2T–V). The anxiety index was higher in SHAM‐hM3Dq rats than in SHAM‐EGFP rats, while the anxiety index was lower in WAS‐hM4Di rats than in WAS‐EGFP rats (Figure 2W).

These data suggest that ACC is crucial for visceral hypersensitivity and anxiety‐like behavior. Activation of ACC^Glu^ resulted in visceral hypersensitivity and anxiety in SHAM rats, whereas suppression of ACC^Glu^ reduced visceral hypersensitivity as well as anxiety in WAS rats.

### 2.3 An Excitatory ACC^Glu^‐ AIC^Glu^ Pathway is Involved in WAS‐Induced Visceral Hypersensitivity and Anxiety‐Like Behavior

**FIGURE 3:**
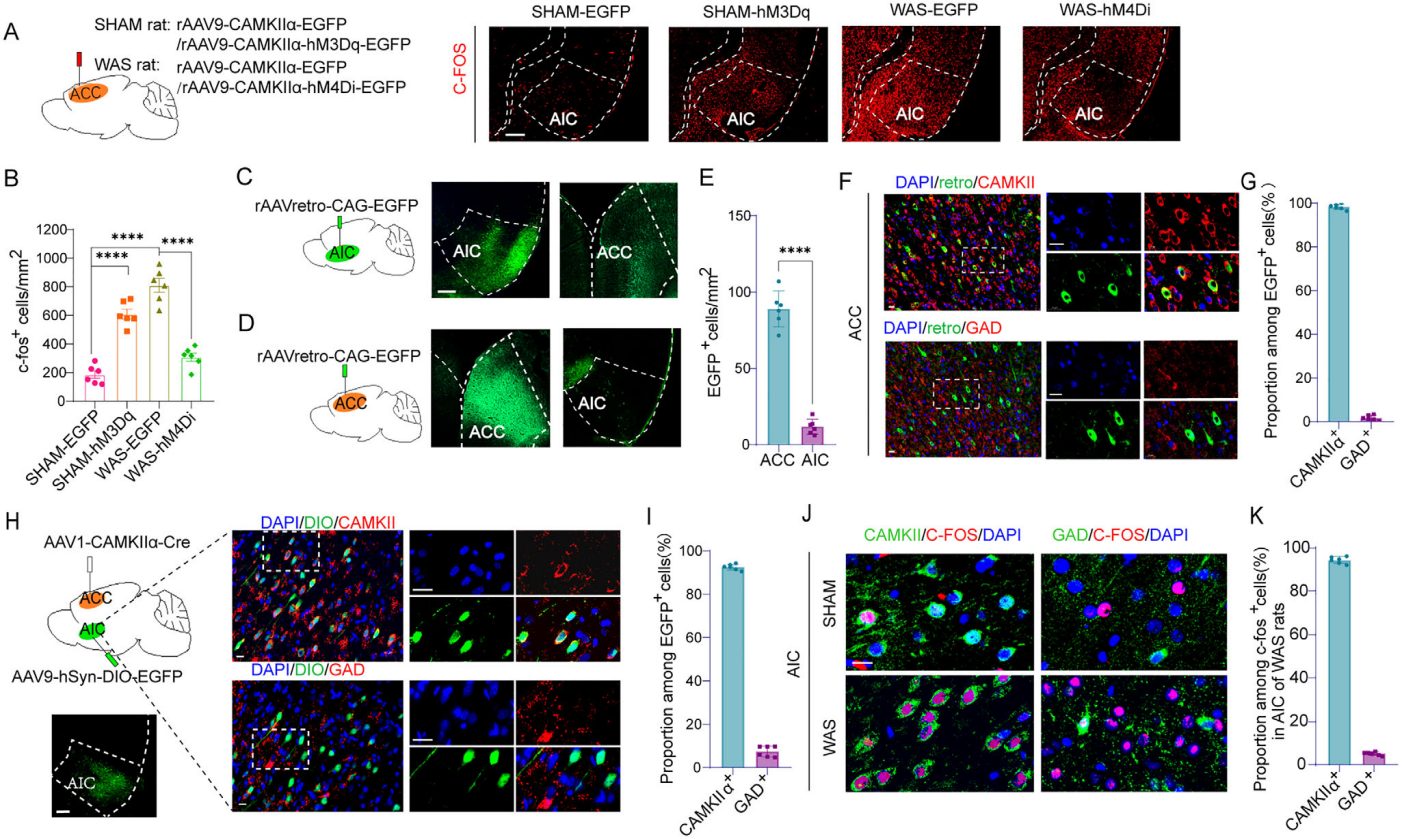
Identification of the excitatory ACC^Glu^‐AIC^Glu^ neural pathway. (A) Schematic of virus injection strategies and typical images of c‐fos expression in the AIC. (B) Quantification of c‐fos expression in the AIC following ACC^Glu^ manipulation. Scale bar, 500 µm (one‐way ANOVA with Tukey's post‐hoc test, B, F _(3, 20)_ = 63.83, *p* < 0.0001). (C,D) Schematic of unilateral retrograde virus injection in the AIC (C) or ACC (D), and the typical images of viral expression, respectively. Scale bar, 500 µm. (E) Comparison of the numbers of EGFP^+^ cells projecting from ACC to AIC and from AIC to ACC (unpaired Student's *t‐*test, E, t _(10)_ = 14.81, *p* < 0.0001). (F,G) Immunostaining against CAMKIIα or GAD65+67 in EGFP‐labeled neurons that had projection to the AIC within the ACC. Representative images of colocalization in (F), statistical results in (G), Scale bar, 20 µm. (H) Left: schematic of Cre/DIO based anterograde trans‐monosynaptic virus tracing strategy (top), as well as typical image of the viral expression within the AIC (bottom). Right: images showing immunostaining against CAMKIIα or GAD65+67 in AIC EGFP‐labeled neurons that received anterograde trans‐monosynaptic virus from ACC. Scale bar, 20 µm. (I) Statistical results of the colocalization of AIC EGFP‐labeled neurons that received anterograde trans‐monosynaptic virus from ACC with CAMKIIα or GAD65+67. (J,K) Immunostaining against CAMKIIα or GAD65+67 in c‐fos positive neurons in the AIC of WAS and SHAM rats. Representative images of colocalization in (J), statistical results in (K), Scale bar, 20 µm. Data presented as mean ± SEM (*n* = 6 rats). ****p < 0.0001.

As two pivotal nodes within SN, AIC and ACC are found to be co‐activated in fMRI studies examining pain or psychological distress [20]. To elucidate the interrelationship between ACC and AIC in the context of WAS‐induced visceral hypersensitivity and anxiety‐like behavior, we selectively modulated the activity of ACC^Glu^ and subsequently analyzed the activity within the AIC using c‐fos immunostaining as a marker of neuronal activation. The results showed that bilateral activation of ACC^Glu^ via hM3Dq in SHAM rats increased AIC c‐fos expression, while inhibition of ACC^Glu^ neurons via hM4Di in WAS rats resulted in the reduction of AIC c‐fos expression (Figure 3A,B). Furthermore, WAS‐EGFP rats showed more c‐fos‐positive cells in AIC than SHAM‐EGFP rats (Figure 3A,B). These findings indicate that activation of AIC neurons, which involved in WAS‐induced comorbid visceral hypersensitivity and anxiety, is modulated by the activity of ACC.

Next, we observed anatomical connections between the ACC and AIC regions of rats. A retrograde virus (rAAVretro‐CAG‐EGFP) was micro‐injected into the unilateral AIC region (Figure 3C, left and middle). Four weeks after virus injection, a substantial population of neurons in the ipsilateral ACC region projecting to AIC was identified by EGFP marker (Figure 3C, right). In addition, the same retrograde virus was also micro‐injected into the unilateral ACC region (Figure 3D, left and middle), but only exhibited a weak projection from AIC to the ipsilateral ACC (Figure 3D right, E). Further immunostaining in ACC, which received retrograde virus from AIC, showed that these neurons projecting to AIC were CAMKIIα‐positive glutamatergic neurons rather than GAD65+67‐positive GABAergic neurons (Figure 3F,G). To study the types of AIC neurons that received ACC glutamatergic projections, a Cre recombinase/double‐floxed inverted open reading frame (Cre/DIO) based anterograde trans‐monosynaptic system was applied. rAAV1‐CAG‐Cre was injected into ACC, and rAAV9‐hSyn‐DIO‐EGFP was injected into the ipsilateral AIC (Figure 3H). Double‐label fluorescence showed that most of the neurons in AIC receiving projection from ACC were CAMKIIα‐positive glutamatergic neurons (Figure 3H,I). Additionally, the activated neurons in AIC of WAS rats were found to be CAMKIIα‐positive glutamatergic neurons rather than GAD65+67‐positive GABAergic neurons (Figure 3J,K).

**FIGURE 4:**
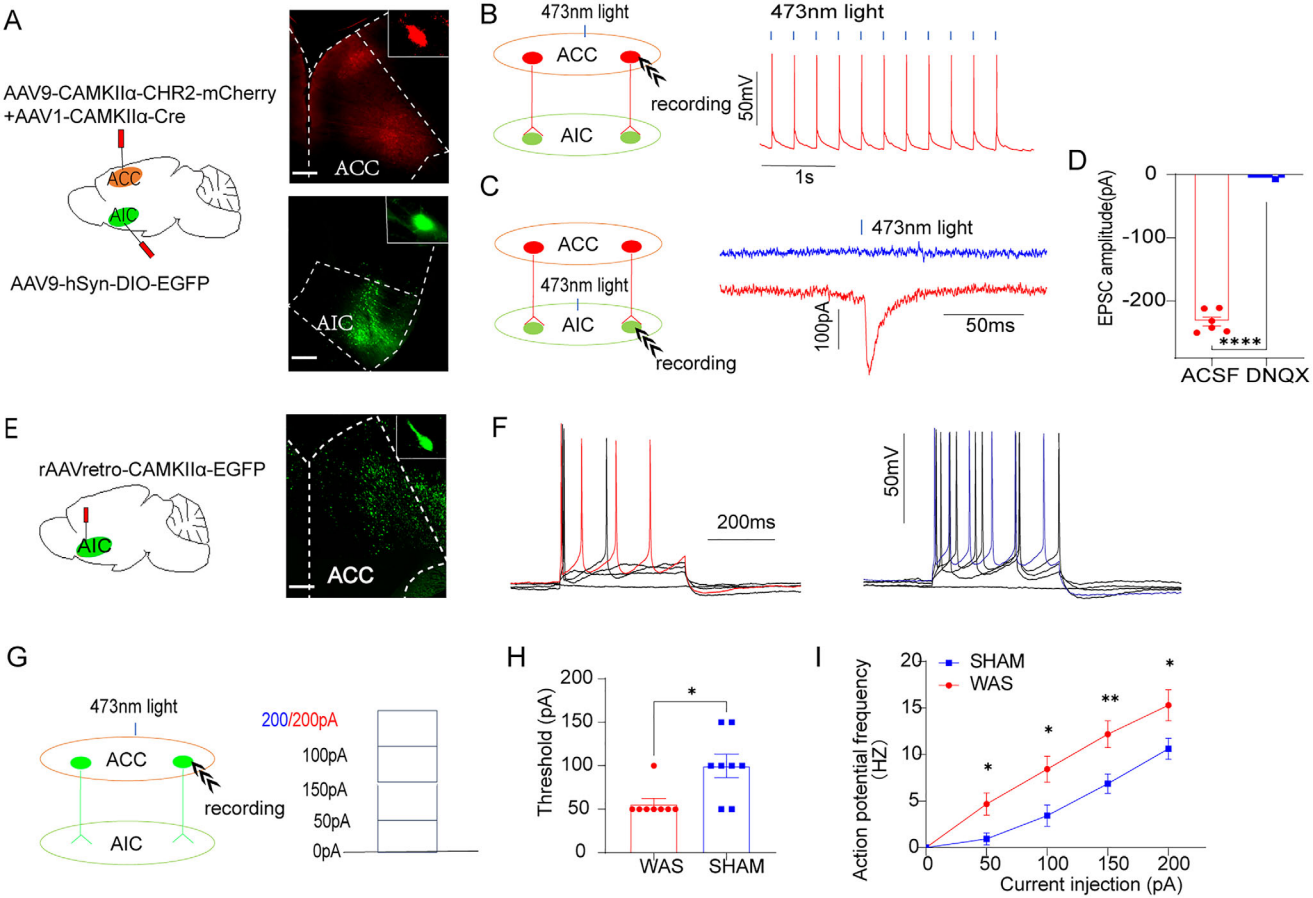
Involvement of the excitatory ACC‐AIC pathway in WAS‐induced visceral hypersensitivity and anxiety‐like behaviors. (A) Left: schematic of optogenetics strategy combined with Cre/DIO based anterograde trans‐monosynaptic virus tracing strategy. Right: expression of optogenetic virus in the ACC (top) and anterograde trans‐monosynaptic virus in the AIC (bottom). Scale bar, 500 µm. (B) Left: schematic of the recording configuration in ACC slices. Right: sample traces of action potentials (APs) evoked by light (473 nm, 15 ms, blue line) recorded in ACC mCherry‐positive neurons. (C,D) Light‐evoked (473 nm, 15 ms, blue line) excitatory postsynaptic current (EPSC) in AIC neurons before and after DNQX (10 µM) treatment. Schematic of the recording configuration and representative traces of EPSC in (C), summarized data in (D) (paired Student's *t‐*test, (D), t _(5)_ = 29.25, *p* < 0.0001). (E–I) Excitability of ACC^Glu^ neurons that have projection to the AIC. Schematic of retrograde virus injection in the AIC and typical image showing the viral expression in ACC cells in (E), schematic of the recording configuration and protocol of injected currents in (F), raw traces in (G) (Red or blue trace is 200 pA current‐induced APs), statical analysis of minimal injected current to induce APs in (H), statical analysis of frequency of induced APs at different current steps in (I). Scale bar, 500 µm (Mann Whitney test, H, *p* = 0.0249; unpaired Student's *t‐*test, I, 50pA, t _(14)_ = 2.743, *p* = 0.0159; 100pA, t _(14)_ = 2.744, *p* = 0.0158; 150pA, t _(14)_ = 2.985, *p* = 0.0098; 200pA, t _(14)_ = 2.326, *p* = 0.0355). Data presented as mean ± SEM (*n* = 6 cells from 3 rats for D, *n* = 8 cells from 4 rats for H). *p < 0.05, **p < 0.01, ***p < 0.001, ****p < 0.0001.

In vitro optogenetics were then performed to study the function of ACC projections to AIC. Meanwhile, to label the AIC neurons receiving ACC fibers, the previously described anterograde trans‐monosynaptic system was also employed. In detail, a combination of rAAV9‐CAG‐channelrhodopsin‐2 (CHR2)‐mCherry and rAAV1‐CAG‐Cre virus were injected into the ACC region. The rAAV9‐hSyn‐DIO‐EGFP virus was injected into the AIC region (Figure 4A, left). The ACC injection sites were confirmed by mCherry‐labeled neurons (Figure 4A, right). Through whole‐cell recording in brain slices, we found that ACC mCherry‐labeled neurons were activated by light exposure (473 nm, 3HZ, blue line) (Figure 4B). Meanwhile, the neurons of AIC that received ACC terminals were marked by EGFP (Figure 4A, right). Whole‐cell recording in neurons in AIC showed that single light pulses (15 ms) induced excitatory postsynaptic currents (oEPSCs) in AIC EGFP‐labeled neurons, where the ACC neuron projection terminated (Figure 4C). The oEPSCs were blocked by the perfusion of α‐amino‐3‐hydroxy‐5‐methyl‐4‐isoxazole‐propionicacid receptor (AMPAR) antagonist 6,7‐dinitroquinoxaline‐2,3‐dione (DNQX), suggesting that the oEPSCs are AMPA currents (Figure 4C,D).

To further investigate the functional alterations of ACC^Glu^ projecting to the AIC, rAAVretro‐ CAMKIIα‐EGFP was injected into AIC of WAS and SHAM rats, respectively (Figure 4E). Using this retrograde virus with EGFP, we labeled ACC^Glu^ neurons that projecting to AIC and selectively recorded their firing activity. Whole‐cell recording showed the threshold current of these ACC^Glu^ neurons was much lower in WAS rats than that in SHAM rats (Figure 4F–H). In addition, firing frequency of these neurons in WAS rats was higher than in SHAM rats following injections of 50 to 200 pA current (Figure 4G,I).

These results suggest a direct monosynaptic excitatory projection from ACC^Glu^ to AIC^Glu^, with the enhanced activity in WAS‐induced comorbid visceral hypersensitivity and anxiety.

### 2.4 Chemogenetic Activation of the ACC‐AIC Circuit Directly Triggers Visceral Hypersensitivity and Anxiety‐Like Behavior

**FIGURE 5:**
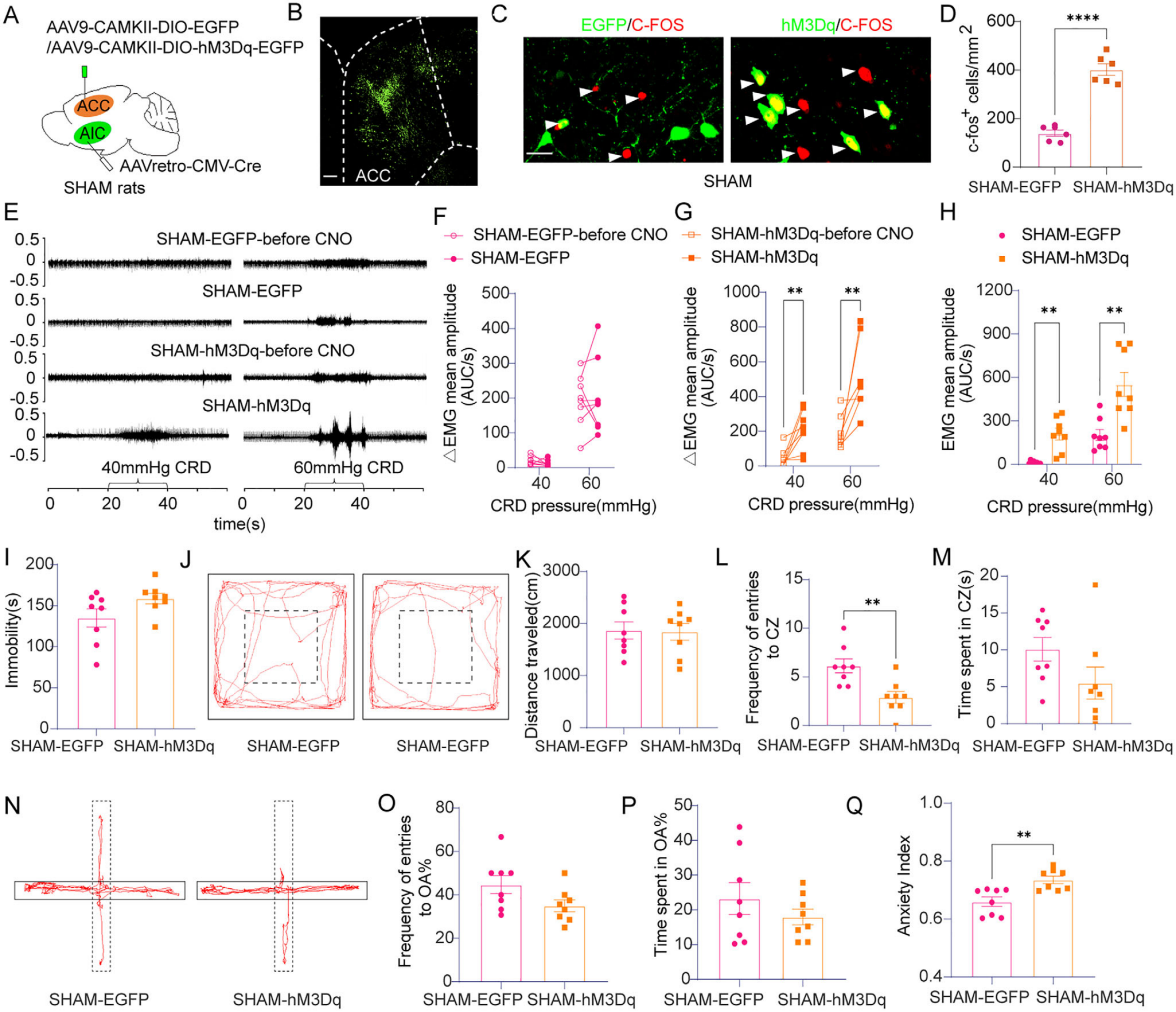
Chemogenetic activation of ACC‐AIC circuit induces visceral hypersensitivity and anxiety‐like behaviors. (A) Schematic of bilateral anterograde‐DIO virus injections in ACC as well as retrograde‐CRE virus injections in AIC of SHAM rats. (B) Representative image of ACC injection sites. Scale bar, 200 µm. (C,D) C‐fos expression in ACC neurons post‐virus expression. Representative images of colocalization in (C), statistical results in (D), Scale bar, 20 µm (unpaired Student's *t*‐test, D, t _(10)_ = 9.677, *p* < 0.0001). (E–H) Activation of ACC‐AIC circuit triggers visceral hypersensitivity. Representative external abdominal oblique muscle EMG recordings in (E), analysis of the mean amplitude of △EMG in (F–H) (paired Student's *t*‐test, F, 40mmHg, t _(7)_ = 1.323, *p* = 0.2275; 60mmHg, t _(7)_ = 0.3050, *p* = 0.7692; G, 60mmHg, t _(7)_ = 3.786, *p* = 0.0068; Wilcoxon matched‐pairs signed rank test, G, 40mmHg, *p* = 0.0078; unpaired Student's *t‐*test, H, 40mmHg, t _(7.107)_ = 4.867, *p* = 0.0017; 60mmHg, t _(14)_ = 3.874, *p* = 0.0017). (I) Immobility time during FST (unpaired Student's *t*‐test, t _(14)_ = 1.860, *p* = 0.084). (J–M) Results of OFT. Representative exploration traces in (J), statistical results of total distance in (K), analysis of frequency of entries into the CZ in (L), analysis of time spent in the CZ in (M) (unpaired Student's *t*‐test, K, t _(14)_ = 0.1218, *p* = 0.908; (L, t _(14)_ = 3.448, *p* = 0.0039; Mann Whitney test, M, *p* = 0.830). (N–Q) Results of EPM. Representative exploration traces in (N), analysis of percentage of entries into OA in (O), analysis of percentage of time spent in the OA in (P), statistical results of the Anxiety index in (Q) (unpaired Student's *t*‐test, O, t _(14)_ = 1.961, *p* = 0.0701; P, t _(14)_ = 1.041, *p* = 0.3154; Mann Whitney test, Q, *p* = 0.0051). Data presented as mean ± SEM (*n* = 6 rats for (D), *n* = 8 rats for (E–Q). **p < 0.01, ***p < 0.001.

Based on the observation of elevated activity in ACC‐projecting neurons within the AIC during WAS‐induced visceral hypersensitivity and anxiety‐like behavior, we hypothesized that the selective activation of excitatory input from ACC to AIC may contribute to the manifestation of WAS‐induced behaviors. To test this hypothesis, we injected rAAV9‐CAMKIIα‐hM3Dq‐DIO‐EGFP (referred to as the SHAM‐hM3Dq group) or rAAV9‐CAMKIIα‐DIO‐EGFP (referred to as the SHAM‐EGFP group) into ACC bilaterally. Concurrently, rAAVretro‐CMV‐Cre was injected into AIC of these rats to selectively target the ACC^Glu^ neurons that projected to AIC (Figure 5A). The expression of the virus was confirmed to be predominantly localized to neurons in the ACC region (Figure 5B). Upon administration of CNO, a significant increase in c‐fos‐positive cells within the ACC of rats in the SHAM‐hM3Dq group was found, indicating the cellular activation mediated by hM3Dq (Figure 5C,D). Moreover, CNO injection increased VMRs in SHAM‐hM3Dq rats to both 40 and 60mmHg CRD stimulation, while did not change the VMRs to both 40 and 60mmHg CRD stimulation in rats from the SHAM‐EGFP (Control) group (Figure 5E–G). The comparation between SHAM‐EGFP and SHAM‐hM3Dq rats showed that activation of the ACC^Glu^‐AIC pathway effectively induced visceral hypersensitivity (Figure 5H).

Behavior tests were conducted to figure out the role of the ACC‐AIC circuit in emotion regulation. FST and OFT showed that activation of the ACC‐AIC pathway failed to regulate the depression‐like behavior and the locomotion of SHAM rats (Figure 5I–K). Moreover, in the OFT, while the number of entries into the CZ was reduced following ACC‐AIC circuit activation, there was no significant change in the duration spent within the CZ (Figure 5L,M). For the EPM, although there was no difference in the frequency of OA entries and the time spent in the OA between the SHAM‐hM3Dq and SHAM‐EGFP groups, the anxiety index was higher in the SHAM‐hM3Dq rats compared with the SHAM‐EGFP rats following CNO injection (Figure 5N–Q). This increase in the anxiety index suggests that selective activation of the ACC‐AIC circuit contributes to enhanced anxiety‐like behavior.

### 2.5 Chemogenetic Inhibition of ACC‐AIC Projection Ameliorates WAS‐Induced Visceral Hypersensitivity and Anxiety‐Like Behaviors

**FIGURE 6:**
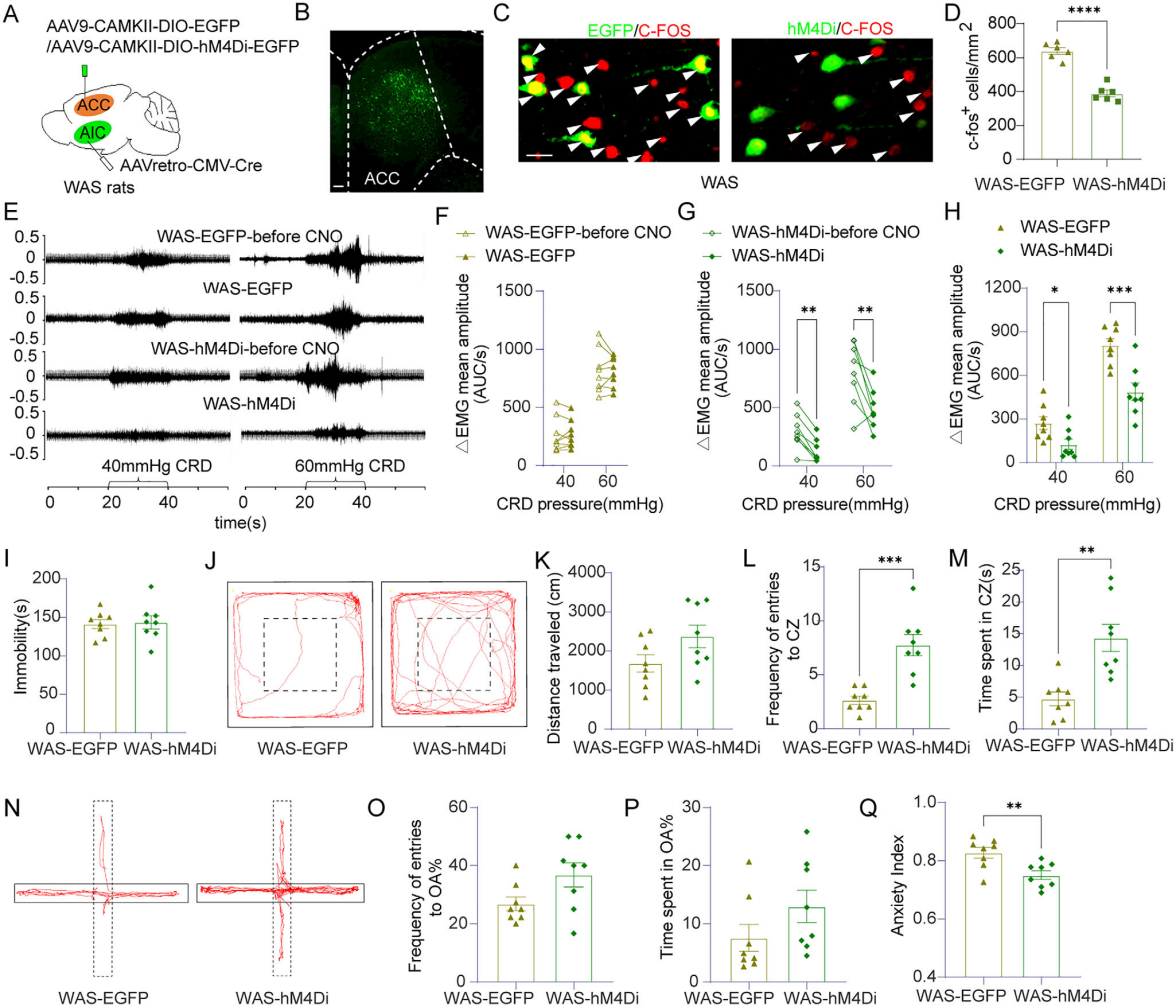
Chemogenetic Inhibition of ACC‐AIC projection ameliorates WAS‐induced visceral hypersensitivity and anxiety‐like behaviors. (A) Schematic of bilateral anterograde‐DIO virus injections in ACC as well as retrograde‐CRE virus injections in AIC of WAS rats. (B) Representative image of ACC‐injection sites. Scale bar, 200 µm. (C,D) C‐fos expression in ACC neurons post‐virus expression. Representative images of colocalization in (C), statistical results in (D), Scale bar, 20 µm (unpaired Student's *t*‐test, D, t _(10)_ = 9.156, *p* < 0.0001). (E–H) Inhibition of the ACC‐AIC circuit ameliorates visceral hypersensitivity induced by WAS. Representative external abdominal oblique muscle EMG recordings in (E), analysis of the mean amplitude of △EMG in (F–H) (paired Student's *t*‐test, F, 40mmHg, t _(7)_ = 0.1729, *p* = 0.8676; 60mmHg, t _(7)_ = 0.3209, *p* = 0.7576; G, 40mmHg, t _(7)_ = 6.049, *p* = 0.0005; 60mmHg, t _(7)_ = 3.590, *p* = 0.0089; unpaired Student's *t*‐test, H, 40mmHg, t _(14)_ = 2.632, *p* = 0.0197; 60mmHg, t _(14)_ = 4.257, *p* = 0.0008). (I) Immobility time during FST (unpaired Student's *t*‐test, t _(14)_ = 0.2040, *p* = 0.8413). (J–M) Results of OFT. Representative exploration traces in (J), statistical results of total distance in (K), analysis of frequency of entries into the CZ in (L), analysis of time spent in the CZ in (M) (unpaired Student's *t*‐test, K, t _(14)_ = 1.896, *p* = 0.0788; L, t _(4.895)_ = 9.017, *p* = 0.0008; M, t _(14)_ = 4.020, *p* = 0.0013). (N–Q) Results of EPM. Representative exploration traces in (N), analysis of percentage of entries into OA in (O), analysis of percentage of time spent in the OA in (P), statistical results of the Anxiety index in (Q) (unpaired Student's *t*‐test, O, t _(14)_ = 2.080, *p* = 0.0564; Q, t _(14)_ = 3.248, *p* = 0.0058; Mann Whitney test, P, *p* = 0.083). Data presented as mean ± SEM (*n* = 6 rats for (D), *n* = 8 rats for (E–Q). *p < 0.05, **p < 0.01, ***p < 0.001.

The above results suggest that activation of the ACC‐AIC pathway facilitates visceral pain and anxiety. Therefore, to investigate whether inhibition of the ACC‐AIC projection could ameliorate WAS‐induced colorectal pain and anxiogenic effects, rAAV9‐CAMKIIα‐hM4Di‐DIO‐EGFP (WAS‐hM4Di group) or rAAV9‐CAMKIIα‐DIO‐EGFP (WAS‐EGFP group) was injected into the ACC of WAS rats. Simultaneously, rAAVretro‐CMV‐Cre was injected into AIC of these rats to selectively modulate the ACC^Glu^ neurons that projected to AIC (Figure 6A). The virus was confirmed to be expressed in the neurons of the ACC region (Figure 6B). Following CNO administration, rats in the WAS‐hM4Di group exhibited less c‐fos‐positive cells compared with those in the WAS‐EGFP group (Figure 6C,D). After CNO injection, VMRs in WAS‐EGFP rats remained unchanged (Figure 6E,F), whereas VMRs in WAS‐hM4Di rats to both 40 and 60mmHg CRD stimulation decreased remarkably compared with their baseline levels (Figure 6E,G). Comparative analysis between WAS‐EGFP and WAS‐hM4Di rats showed that ACC‐AIC pathway inhibition alleviated visceral hypersensitivity of WAS rats (Figure 6H).

To evaluate the effects of ACC‐AIC pathway inhibition on WAS‐induced anxiety, behavior tests were performed. FST and OFT showed that the depression‐like behavior and the locomotor activity of WAS rats were unaffected by ACC‐AIC pathway inhibition (Figure 6I–K). However, suppressing the ACC‐AIC circuit enhanced rats' entries into and residence time in CZ in OFT (Figure 6L,M). Additionally, although the increase in OA entries and time spent in OA did not have statistical significance, inhibition of the ACC‐AIC circuit significantly diminished the anxiety index in EPM for WAS‐hM4Di rats (Figure 6N–Q). These data suggest that inhibition of ACC glutamatergic inputs to AIC attenuates visceral hypersensitivity and anxiety‐like behavior.

### 2.6 Bidirectional Modulation of AIC^Glu^ Activity Could Mimic or Reverse the WAS‐Induced Visceral Hypersensitivity and Anxiety‐Like Behaviors

**FIGURE 7:**
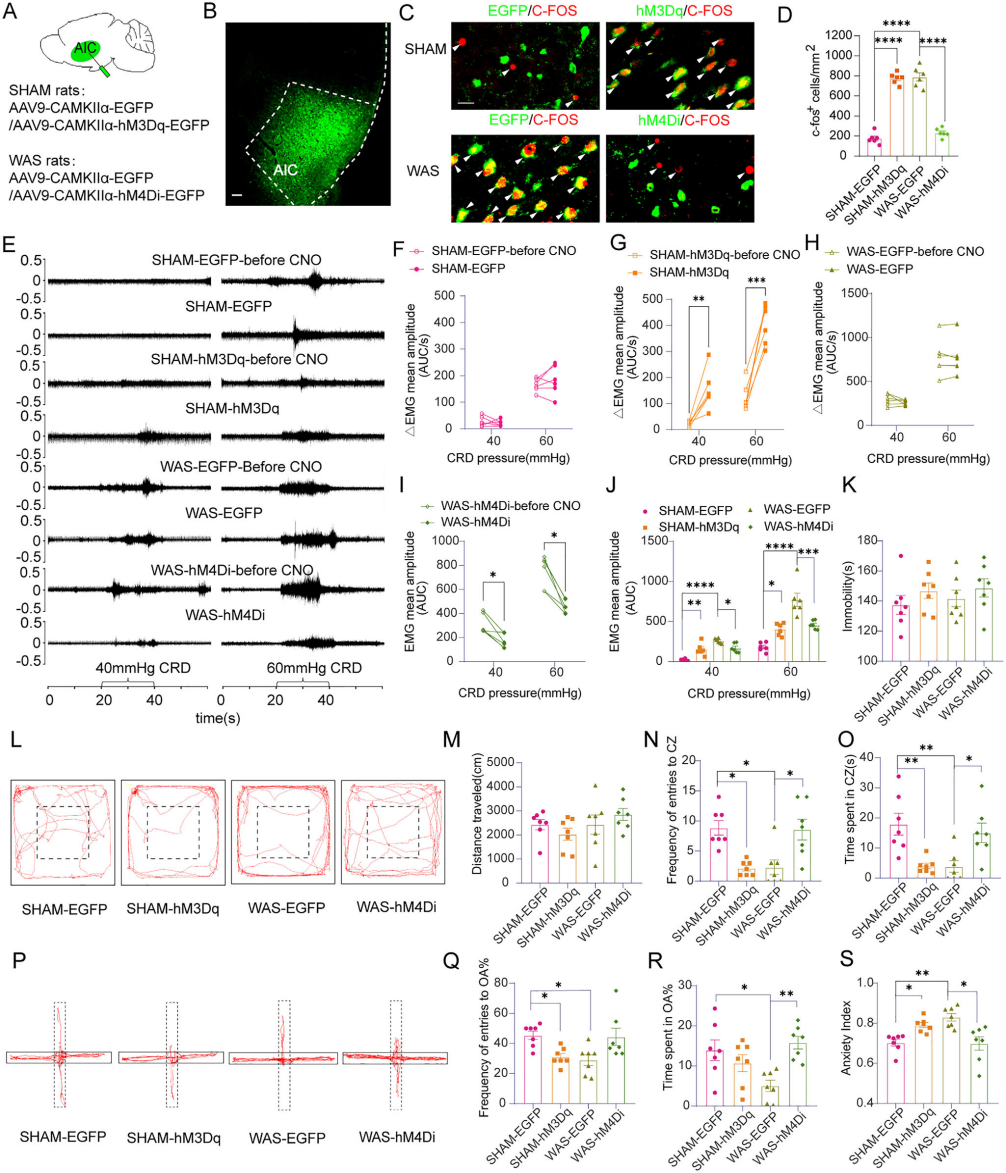
Bidirectional modulation of AIC^Glu^ activity mimics or reverses the WAS‐induced visceral hypersensitivity and anxiety‐like behaviors. (A) Schematic of bilateral rAAV virus injections in AIC of WAS or SHAM rats. (B) Representative image of AIC‐injection sites. Scale bar, 200 µm. (C,D) C‐fos expression in AIC neurons post‐virus expression. Representative images of colocalization in C, statistical results in D, Scale bar, 20 µm (one‐way ANOVA with Tukey's post‐hoc test, D, F _(3, 20)_ = 143.2, *p* < 0.0001). (E–J) Activation of AIC^Glu^ mimicked visceral hypersensitivity, whereas suppression of AIC^Glu^ reversed the visceral hypersensitivity induced by WAS. Representative external abdominal oblique muscle EMG recordings in E, analysis of the mean amplitude of △EMG in F‐J (paired Student's *t*‐test, F, 40mmHg, t _(5)_ = 0.6127, p = 0.5669; 60mmHg, t _(5)_ = 1.003, p = 0.3617; G, 40mmHg, t _(5)_ = 4.302, *p* = 0.0077; 60mmHg, t _(5)_ = 8.465, *p* = 0.0004; H, 40mmHg, t _(5)_ = 1.319, *p* = 0.2443; 60mmHg, t _(5)_ = 0.0675, *p* = 0.9488; Wilcoxon matched‐pairs signed rank test, I, 40mmHg, *p* = 0.0313; 60mmHg, *p* = 0.0313; one‐way ANOVA with Tukey's post‐hoc test, J, 40mmHg, F _(3, 20)_ = 22.74, *p* < 0.0001; 60mmHg, F _(3, 20)_ = 26.14, *p* < 0.0001). (K) Immobility time during FST (one‐way ANOVA with Tukey's post‐hoc test, F _(3, 24)_ = 0.7811, *p* = 0.5161). (L‐O) Results of OFT. Representative exploration traces in L, statistical results of total distance in M, analysis of frequency of entries into the CZ in N, analysis of time spent in the CZ in O (one‐way ANOVA with Tukey's post‐hoc test, M, F _(3, 24)_ = 1.347, *p* = 0.2828; O, F _(3, 24)_ = 7.594, *p* = 0.0010; Kruskal‐Wallis test with Dunn's post‐hoc test, N, *p* = 0.0014). (P‐S) Results of EPM. Representative exploration traces in P, analysis of percentage of OA in Q, analysis of percentage of time spent in the OA in R, statistical results of the Anxiety index in S (Kruskal‐Wallis test with Dunn's post‐hoc test, Q, *p* = 0.0037; S, *p* = 0.0004; one‐way ANOVA with Tukey's post‐hoc test, R, F _(3, 24)_ = 5.677, *p* = 0.0044). Data presented as mean ± SEM (*n* = 6 rats for D‐K, *n* = 7 rats for L‐S). *p < 0.05, **p < 0.01, ***p < 0.001, ****p < 0.0001.

The above experiments suggest that the ACC^Glu^ and ACC‐AIC pathway is involved in the development of WAS‐induced visceral pain and anxiety. To evaluate the contribution of AIC, chemogenetic tools were used in glutamatergic neurons in AIC in WAS model. The rAAV9‐CAMKIIα‐hM3Dq or rAAV9‐CAMKIIα‐EGFP was infused into AIC of SHAM rats, while the rAAV9‐CAMKIIα‐hM4Di or rAAV9‐CAMKIIα‐EGFP was infused into AIC of WAS rats (Figure 7A,B). Subsequent intraperitoneal injection of CNO selectively activated or inhibited the AIC glutamatergic neurons (Figure 7C,D). Similarly, the enhancement of AIC^Glu^ activity resulted in visceral hypersensitivity in SHAM rats, whereas the inhibition of AIC^Glu^ activity ameliorated visceral hypersensitivity in WAS rats (Figure 7E–J).

Behavioral tests revealed that modulation of rats’ AIC^Glu^ activity did not significantly influence the depression‐like behavior as measured by FST (Figure 7K). OFT showed the locomotion was unaffected by modulation of rats’ AIC^Glu^ activity, either (Figure 7L,M). However, in OFT, the enhancement of AIC^Glu^ activity reduced entries into and residence time in CZ in SHAM rats, whereas the inhibition of AIC^Glu^ activity increased entries into and residence time in CZ in WAS rats (Figure 7N,O). For EPM, the activation of AIC^Glu^ led to a reduction in the number of OA entries and an increase in the anxiety index in SHAM rats, while the suppression of AIC^Glu^ increased the time spent in OA and decreased the anxiety index in WAS rats (Figure 7P–S).

These results suggest that activation of AIC^Glu^ mimics WAS‐induced visceral hypersensitivity and anxiety‐like behaviors, whereas inhibition of AIC^Glu^ reverses visceral hypersensitivity and anxiety‐like behaviors induced by WAS.

### 2.7 ACC^Glu^‐AIC^Glu^ Pathway Regulates Expressions of iGluRs in Synaptosomes in AIC

**FIGURE 8:**
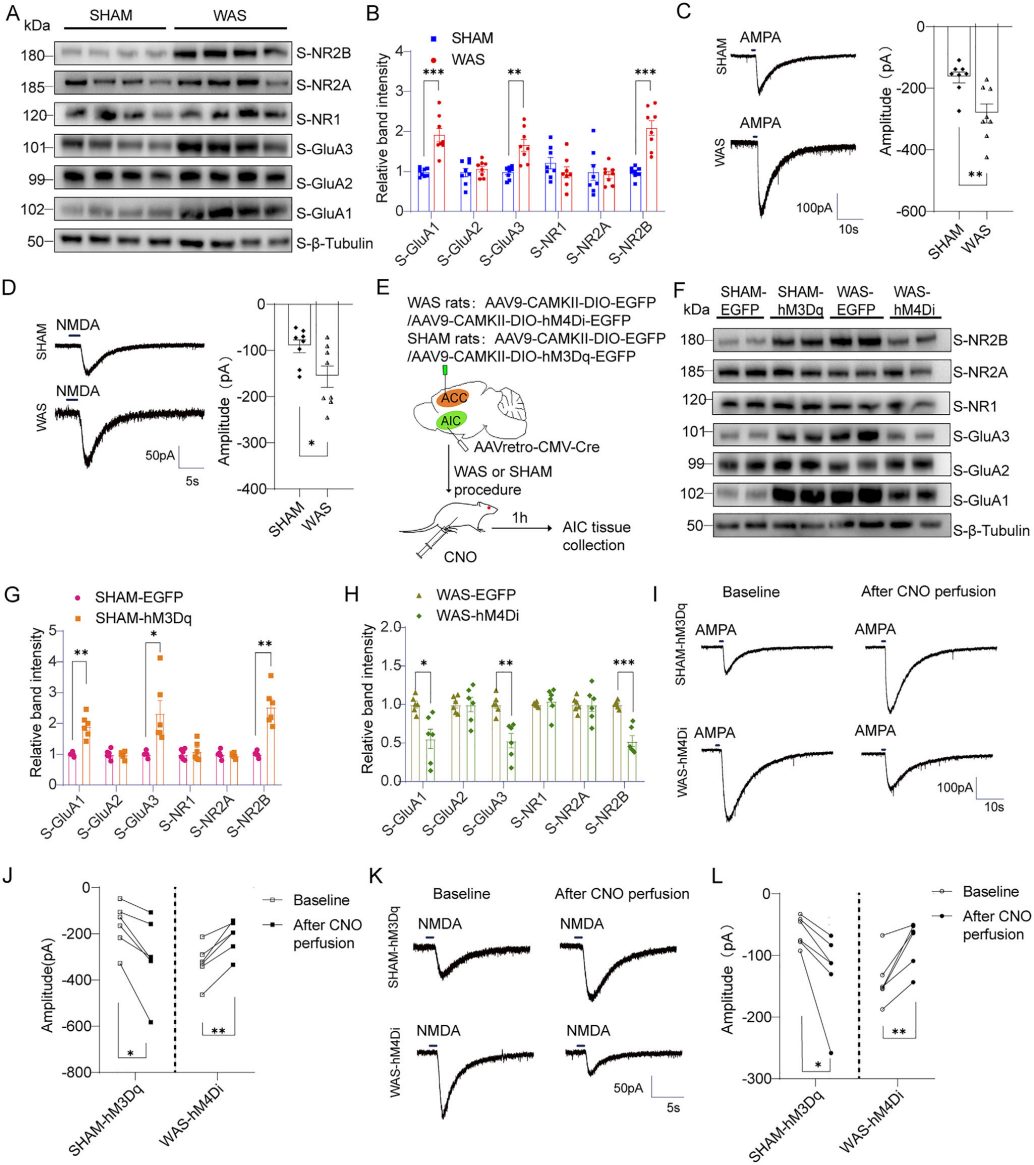
ACC^Glu^‐AIC^Glu^ pathway regulates expressions of iGluRs in synaptosomes in AIC. (A,B) Synaptosomal (‘S’) protein levels of AMPA and NMDA receptors in AIC of WAS and SHAM rats. Representative Western blots in (A), quantification in (B) (unpaired Student's *t*‐test, b, S‐GluA1, t _(7.462)_ = 5.446, *p* = 0.0006; S‐GluA2, t _(14)_ = 0.6821, *p* = 0.5063; S‐GluA3, t _(8.838)_ = 4.264, *p* = 0.0022; S‐NR1, t _(14)_ = 1.178, *p* = 0.2585; S‐NR2A, t _(9.520)_ = 0.2714, *p* = 0.7919; S‐NR2B, t _(8.095)_ = 5.871, *p* = 0.0004). (C) Representative traces(left) and summary data(right) show currents elicited by puff AMPA in AIC neurons from WAS and SHAM rats (unpaired Student's *t*‐test, t _(14)_ = 3.304, *p* = 0.0052). (D) Representative traces(left) and summary data(right) show currents elicited by puff NMDA in AIC neurons from WAS and SHAM rats (unpaired Student's *t*‐test, t _(14)_ = 2.431, *p* = 0.0291). (E) Schematic of chemogenetic modulation and tissue collection of WAS or SHAM rats. (F–H) Synaptosomal protein levels of AMPA and NMDA receptors in AIC after chemogenetic modulation of ACC‐AIC pathway. Representative Western blots in (F), quantification in (G,H) (unpaired Student's *t*‐test, G, S‐GluA1, t _(5.271)_ = 6.186, *p* = 0.0013; S‐GluA2, t _(10)_ = 0.1878, *p* = 0.8548; S‐GluA3, t _(5.088)_ = 3.204, *p* = 0.0233; S‐NR1, t _(10)_ = 0.7248, *p* = 0.4852; S‐NR2A, t _(10)_ = 0.7151, *p* = 0.4909; S‐NR2B, t _(5.224)_ = 6.172, *p* = 0.0014; H, S‐GluA1, t _(6.184)_ = 3.342, *p* = 0.0149; S‐GluA2, t _(10)_ = 0.0036, *p* = 0.9972; S‐GluA3, t _(10)_ = 4.434, *p* = 0.0013; S‐NR1, t _(10)_ = 0.6278, *p* = 0.5442; S‐NR2A, t _(10)_ = 0.0295, *p* = 0.9771; S‐NR2B, t _(5.786)_ = 6.411, *p* = 0.0008). (I,J) Currents elicited by puff AMPA before and after chemogenetic modulation of the ACC‐AIC pathway. Representative traces in (I), quantification in (J) (paired Student's *t*‐test, J, SHAM‐hM3Dq, t _(5)_ = 4, *p* = 0.0103; WAS‐hM4Di, t _(5)_ = 6.676, *p* = 0.0011). (K,L) Currents elicited by puff NMDA before and after chemogenetic modulation of the ACC‐AIC pathway. Representative traces in (K), quantification in (L) (paired Student's *t*‐test, L, SHAM‐hM3Dq, t _(5)_ = 3.251, *p* = 0.0227; WAS‐hM4Di, t _(5)_ = 4.657, *p* = 0.0055). Data presented as mean ± SEM (*n* = 8 rats for B, *n* = 8 cells from 3 rats for C and D, *n* = 6 rats for F and H, *n* = 6 cells from 3 rats for I‐L). *p < 0.05, **p < 0.01, ***p < 0.001.

Given the pivotal role of iGluRs, especially AMPAR and N‐methyl‐D‐aspartate receptor (NMDAR), in excitatory neurotransmission in the central nervous system, we further analyzed the mRNA and protein expression levels of NMDARs (including NR1, NR2A, and NR2B) and AMPARs (including GluA1, GluA2, and GluA3) in AIC via qPCR and western blot. The mRNA (Figure S1A) and total(‘T’) protein levels of GluA2 were reduced in WAS rats compared with those in SHAM rats (Figure S1B,C). Moreover, NMDARs and AMPARs can be categorized into synaptic and extra‐synaptic receptors according to their location. Synaptic receptors are located in the postsynaptic density and directly involved in excitatory neurotransmission, plasticity, and pro‐survival activity, whereas extra‐synaptic receptors located outside the synapse and primarily serve as a mobile reservoir for synaptic insertion and are related to neuronal damage and cell death [32, 33]. Accordingly, we further examined the synaptosomal(‘S’) expressions of these receptors. The successful enrichment and isolation of the synaptosomal fractions were validated by the distribution of postsynaptic density protein 95(PSD95) and proliferating cell nuclear antigen (PCNA) (Figure S1D). PSD95 was sorted predominantly in the synaptic protein fraction. Conversely, PCNA, a protein localized in the nucleus, was not detected in the synaptic protein fraction (Figure S1D). In the AIC of WAS rats, synaptosomal expressions of GluA1 and GluA3 were upregulated (Figure 8A,B). Concurrently, overexpression of NR2B was also found in synaptosomal fractions of WAS rats (Figure 8A,B). To examine the response of increased synaptosomal iGluRs to AMPA or NMDA in AIC neurons in WAS rats, we measured puff‐evoked AMPAR and NMDAR currents via in vitro whole‐cell recording in AIC slices. It showed that the amplitude of both puff‐evoked AMPA and NMDA currents in the AIC neurons increased in WAS rats compared with those in SHAM rats (Figure 8C,D), indicating enhanced postsynaptic AMPAR and NMDAR activity in the AIC induced by WAS. The results suggest that the number and response of synaptosomal iGluR, GluA1, GluA3, and NR2B, increase in WAS rats.

To observe the effect of ACC‐AIC circuit activation/inhibition on subunits of AMPARs and NMDARs, chemogenetic modulation strategies were employed (Figure 8E). Interventions targeting the ACC‐AIC pathway did not result in alterations in the total expression of NMDARs or AMPARs, including GluA2(Figure S1E–G). However, chemogenetic activation of the ACC‐AIC pathway mimicked the increase of synaptosomal expressions of GluA1, GluA3, and NR2B in AIC of SHAM rats (Figure 8F,G), whereas the inhibition of this pathway led to a restoration of synaptosomal expressions of GluA1, GluA3, and NR2B in AIC of WAS rats (Figure 8F,H). Moreover, in in vitro whole‐cell recordings, it showed that activation of the ACC‐AIC pathway with CNO perfusion enhanced the amplitude of the currents elicited by puff application of AMPA or NMDA in SHAM‐hM3Dq rats. Concurrently, both AMPA and NMDA‐elicited currents in AIC neurons were reduced after inhibition of the ACC‐AIC pathway (Figure 8I–L).

These results suggest the ACC^Glu^‐AIC^Glu^ pathway is critical in modulating synaptosomal expression of GluA1, GluA3 and NR2B in the WAS model, altering postsynaptic AMPAR and NMDAR currents.

### 2.8 Inhibition of GluA1, GluA3 and NR2B in AIC Relieves Visceral Hypersensitivity and Anxiety in WAS Rats

**FIGURE 9:**
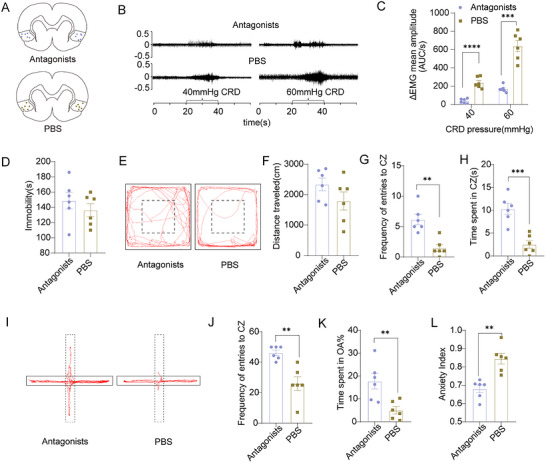
Inhibition of GluA1, GluA3 and NR2B in AIC relieves visceral hypersensitivity and anxiety in WAS rats. (A) The location of cannula placement in different groups. (B,C) Microinjection of antagonists (NASPM and Ro25‐6981) prevents visceral hypersensitivity in WAS rats. Representative external abdominal oblique muscle EMG recordings in (B), analysis of the mean amplitude of △EMG in (C) (unpaired Student's *t*‐test, C, 40mmHg, t _(10)_ = 7.539, *p* < 0.0001; 60mmHg, t _(5.589)_ = 7.425, *p* = 0.0004). (D) Immobility time during FST (unpaired Student's *t*‐test, t _(10)_ = 0.8919, *p* = 0.3934) (). (E–H) Results of OFT. Representative exploration traces in (E), statistical results of total distance in (F), analysis of frequency of entries into the CZ in (G), analysis of time spent in the CZ in (H) (unpaired Student's *t*‐test, F, t _(10)_ = 1.491, *p* = 0.3934; G, t _(10)_ = 4.495, *p* = 0.0012; H, t _(10)_ = 5.248, *p* = 0.0004). (I–L) Results of EPM. Representative exploration traces in I, analysis of percentage of entries into OA in (J), analysis of percentage of time spent in the OA in (K), statistical results of the Anxiety index in (L) (unpaired Student's *t*‐test, J, t _(10)_ = 4.190, *p* = 0.0019; K, t _(10)_ = 3.391, *p* = 0.0069; L, t _(10)_ = 4.573, *p* = 0.0010). Data presented as mean ± SEM (*n* = 6 rats). ** *p* < 0.01, ***p < 0.001, ****p < 0.0001.

To investigate the functional role of synaptic GluA1, GluA3 and NR2B aggregation, we bilaterally microinjected a combination of 1‐Naphthylacetyl spermine trihydrochloride (NASPM) and Ro25‐6981 into the AIC of WAS rats. NASPM serves as a selective antagonist of GluA1‐ and GluA3‐ containing calcium‐permeable AMPARs (CP‐AMPARs) which specifically inhibited the aggregation of them on the postsynaptic surface without affecting total protein expression [34], while Ro25‐6981 is a selective and activity‐dependent antagonist specific to the NR2B subunit of NMDA receptors which reduces synaptic NR2B responses [35, 36]. The injection site accuracy was verified by post hoc histological analysis of brain sections (Figure 9A). Compared with rats that received PBS microinjections, the rats with injection of mixed antagonists exhibited significantly lower VMRs to both 40 and 60mmHg CRD (Figure 9B,C). The FST and OFT showed depression‐like behavior and locomotion was unaffected by the inhibition of CP‐AMPARs and NR2B (Figure 9D–F). However, in the OFT, WAS rats with antagonist microinjection travelled more frequently into CZ and spent more time in CZ compared with WAS rats that received PBS microinjection (Figure 9E,G,H). In the EPM, an increase in the number of OA entries and time spent in OA was found in WAS rats with antagonist microinjection (Figure 9I–K). Meanwhile, the anxiety index reduced greatly following antagonist administration the AIC (Figure 9L). These results suggest that upregulation of GluA1/A3 and NR2B in AIC synaptosomes contribute to visceral hypersensitivity and anxiety‐like behaviors caused by WAS.

### 2.9 Inhibition of ACC^Glu^‐AIC^Glu^ Pathway Fails to Alleviate Visceral Hypersensitivity in a Non‐Mental Stress Model

**FIGURE 10:**
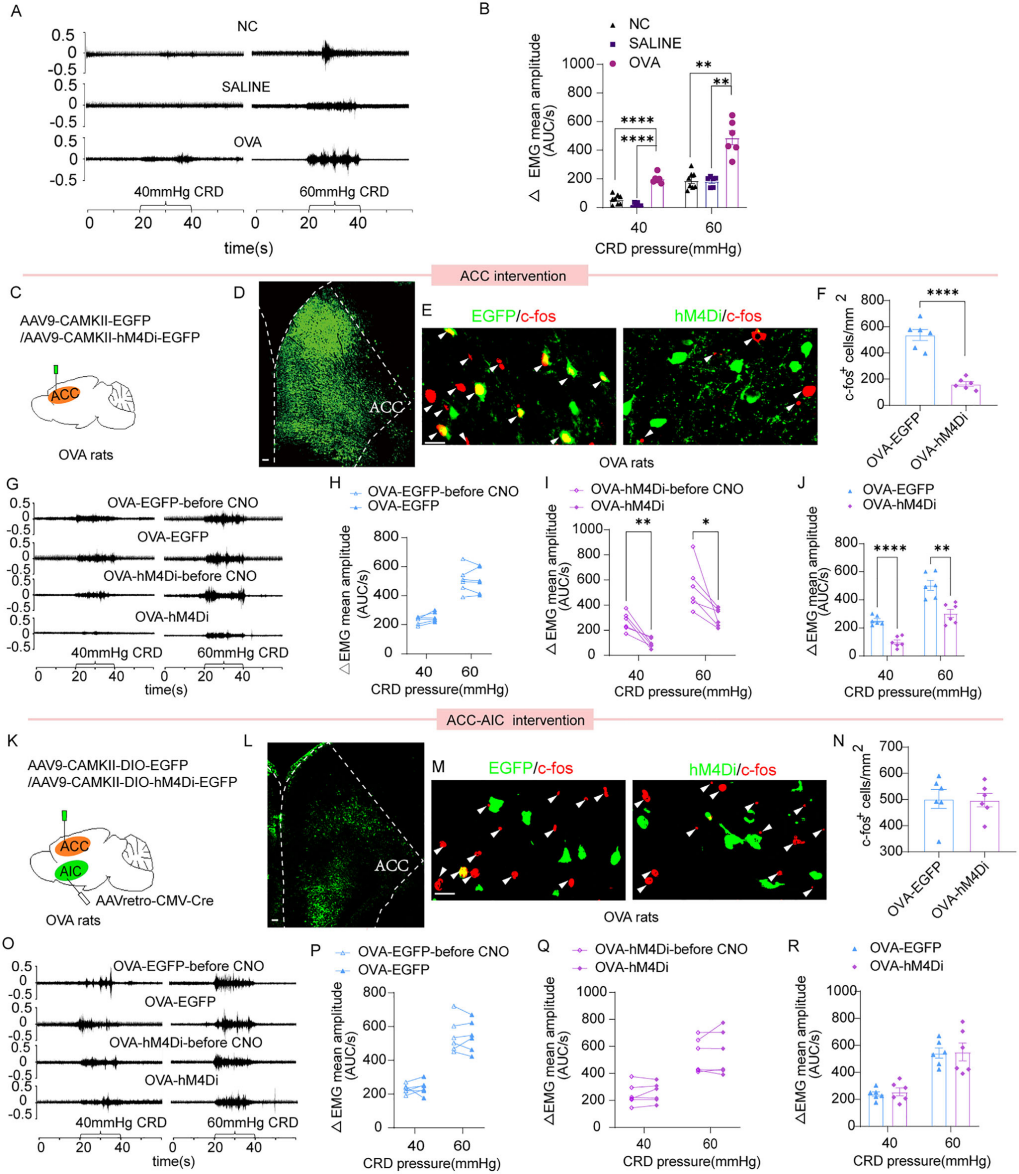
Inhibition of the ACC^Glu^‐AIC^Glu^ pathway fails to alleviate visceral hypersensitivity in a non‐mental stress model. (A,B) VMR to 40 and 60mmHg CRD stimulation. OVA: ovalbumin. Representative external abdominal oblique muscle EMG recordings in (A), analysis of the mean amplitude of △EMG in (B) (one‐way ANOVA test followed by Tukey's post‐hoc test, B, 40mmHg, F _(2, 17)_ = 67.67, *p* < 0.0001; Kruskal–Wallis test followed by Dunnett's post‐hoc test, B, 60mmHg, *p* = 0.0003. (C) Schematic of bilateral rAAV virus injections in ACC of OVA rats. (D) Representative image of ACC injection sites. Scale bar, 200 µm. (E,F) C‐fos expression in ACC post‐virus injection. Representative images of colocalization in (E), statistical results in (F), Scale bar, 20 µm (unpaired Student's *t*‐test, F, t _(10)_ = 8.202, *p* < 0.0001). (G–J) Inhibition of ACC^Glu^ relieves the visceral hypersensitivity induced by OVA. Representative external abdominal oblique muscle EMG recordings in (G), analysis of the mean amplitude of △EMG in (H–J) (Paired Student's *t*‐test, H, 40mmHg, t _(5)_ = 2.347, *p* = 0.0658; 60mmHg, t _(5)_ = 0.2801, *p* = 0.7906; I, 40mmHg, t _(5)_ = 4.486, *p* = 0.0065; 60mmHg, t _(5)_ = 3.139, *p* = 0.0257; Unpaired Student's *t*‐test, J, 40mmHg, t _(10)_ = 7.46, *p* < 0.0001; 60mmHg, t _(10)_ = 4.313, *p* = 0.0015. (K) Schematic of bilateral anterograde‐DIO (in ACC) and retrograde‐CRE (in AIC) virus injections in OVA rats. (L) Representative image of ACC injection sites. Scale bar, 200 µm. (M,N) C‐fos expression in ACC post‐virus injection. Representative images of colocalization in (M), statistical results in (N), Scale bar, 20 µm (unpaired Student's *t*‐test, N, t _(10)_ = 0.1144, *p* = 0.9112). (O‐R) Failure of ACC‐AIC circuit inhibition to reduce OVA‐induced visceral hypersensitivity. Representative external abdominal oblique muscle EMG recordings in (O), analysis of the mean amplitude of △EMG in (P‐R) (paired Student's *t*‐test, P, 40mmHg, t _(5)_ = 0.4976, *p* = 0.6399; 60mmHg, t _(5)_ = 0.2252, *p* = 0.8308; Q, 40mmHg, t _(5)_ = 0.9588, *p* = 0.3817; 60mmHg, t _(5)_ = 0.7854, *p* = 0.4678; unpaired Student's *t*‐test, R, 40mmHg, t _(10)_ = 0.5248, *p* = 0.6111; 60mmHg, t _(10)_ = 0.1117, *p* = 0.9132). Data presented as mean ± SEM (*n* = 8 rats for the NC group and *n* = 6 rats for all other groups). *p < 0.05, **p < 0.01, ***p < 0.001, ****p < 0.0001.

To elucidate the potential contribution of ACC^Glu^‐AIC^Glu^ circuit to visceral hypersensitivity without anxiety, we used ovalbumin (OVA) injection to establish a visceral pain model without psychological stress in this study [37]. The VMRs to 40 or 60mmHg CRD were found higher in OVA‐injected rats compared with that in both SALINE and NC rats (Figure 10A,B). No significant difference among OVA, SALINE, and NC rats has been detected in anxiety or depression‐like behavior. These included measures of total distance and CZ traveling in the OFT, OA exploration or anxiety index in the EPM, and immobility time in the FST (Figure S2A–I).

Subsequently, we conducted chemogenetic modulation of ACC glutamatergic neurons of OVA rats to assess the role of ACC in OVA‐induced visceral hypersensitivity (Figure 10C,D). Chemogenetic inhibition of ACC glutamatergic neurons resulted in a reduction in the number of c‐fos‐positive neurons in OVA‐hM4Di rats (Figure 10E,F), which subsequently led to an alleviation of visceral hypersensitivity (Figure 10G–J) without affecting anxiety or depression‐related behaviors (Figure S3A–H).

Next, the Cre/DIO based virus was injected to selectively inhibit the activation of the ACC^Glu^‐AIC^Glu^ circuit in OVA rats to determine whether this specific circuit was involved in OVA‐induced visceral hypersensitivity (Figure 10K,L). However, the ACC^Glu^‐AIC^Glu^ circuit was not activated in OVA‐EGFP rats, as evidenced by no change in the number of c‐fos positive‐cells in ACC region of OVA‐hM4Di rats with CNO administration (Figure 10M,N). Intraperitoneal administration of CNO failed to elicit a reduction in VMRs in either OVA‐EGFP rats or OVA‐hM4Di rats (Figure 10O–R). In addition, inhibition of the ACC^Glu^‐AIC^Glu^ circuit exerted no influence on anxiety or depression‐related behavior in OVA rats (Figure S4A–H).

These results suggest that activation of ACC^Glu^ is also crucial to visceral hypersensitivity without mental stress, but ACC^Glu^‐AIC^Glu^ circuit activation is not involved.

## 3 Discussion

The high co‐occurrence of chronic visceral pain and anxiety disorders represents a major clinical challenge, yet the neural mechanisms that unify these sensory and affective dimensions remain poorly defined. In the present study, we identified a novel cortical‐cortical projection from ACC^Glu^ to AIC^Glu^ specifically mediated stress‐induced visceral hypersensitivity and anxiety‐related behaviors but not OVA‐induced visceral hypersensitivity in rats. Furthermore, we found elevated synaptosomal expression of GluA1/A3, and NR2B of iGluRs and enhanced postsynaptic NMDAR and AMPAR currents in the AIC of WAS rats, which was alleviated by chemogenetic inhibition of ACC^Glu^‐AIC^Glu^ circuit.

While several brain circuits are associated with visceral pain, the neural circuits underlying the interaction of negative emotions and visceral pain are not well characterized [38, 39, 40, 41, 42]. In humans and rodents, the ACC is a critical region for processing both emotional and sensory components of chronic pain, yet the specific neuronal subtypes involved are largely unidentified [17]. The present study found that ACC glutamatergic rather than GABAergic neurons were activated under prolonged WAS, consistent with rat models of stress‐related neuropathic pain and anxiety‐associated pancreatitis‐induced visceral pain [14, 43]. Moreover, the application of chemogenetic techniques for selective modulation of neurons in ACC further confirmed the importance of activated ACC pyramidal neurons in chronic mental stress‐induced anxiety and visceral sensitization. Notably, a previous study has demonstrated that lesions in ACC also alleviate OVA‐induced visceral hypersensitivity in a non‐mental stress context [44]. To determine whether ACC^Glu^ activation is specifically involved in visceral hypersensitivity induced by mental stress, we also employed the OVA model independent of psychological stress. We found that inhibition of ACC^Glu^ also attenuated visceral hypersensitivity in OVA rats. These findings suggest that the ACC is both necessary and sufficient for the development of visceral hypersensitivity, and ACC glutamatergic neurons are critical in processing visceral information, independent of emotional input.

We previously established the necessity of insular cortex(IC) in stress‐related visceral hypersensitivity through bilateral IC lesions [45]. The IC is one of the largest brain regions in rodents, functionally divided into the anterior (AIC) and posterior part (PIC) along the rostro‐caudal axis [46]. The ACC‐PIC pathway has not been shown to affect anxio‐depressive symptoms in nociceptive pain, whereas AIC is shown to be critical in both anxiety and hyperalgesia in painful chronic pancreatitis [47, 48, 49]. In this study, we specifically examined the role of AIC in stress‐induced comorbid visceral hypersensitivity and anxiety, given its projections to the amygdala and its involvement in emotional awareness [46]. Previous studies have indicated that the AIC and the ACC form integral components of the salience network, and these regions are co‐activated in individuals with chronic pain conditions such as IBS [21, 22]. Our findings in the present study demonstrated that AIC glutamatergic neuron activity was modulated by glutamatergic transmission from the ACC.

Our study identified ACC‐AIC connecting neurons in rats, which is consistent with previous tracing studies [27, 28, 29]. More importantly, our anatomical and functional experiments in the current study revealed that the ACC‐AIC connection in rats is predominantly mediated by excitatory projections from ACC to AIC glutamatergic neurons. Chemogenetic activation of the ACC^Glu^‐AIC^Glu^ circuit or AIC^Glu^ alone was sufficient to induce anxiety and visceral hypersensitivity. Conversely, the inhibition of ACC glutamatergic neurons projecting to AIC or the suppression of AIC^Glu^ ameliorated anxiety and visceral hypersensitivity in rats under chronic psychological stress. These results support the hypothesis that the AIC integrates top‐down predictions from higher‐order brain regions, such as ACC, and subsequently conveys descending interoceptive predictions to visceral systems [50].

This study revealed, an intriguing dissociation: ACC inhibition alleviated visceral hypersensitivity in both the OVA and WAS models, whereas selective suppression of the ACC^Glu^‐AIC^Glu^ circuit did not alleviate visceral hypersensitivity induced by OVA injection. This distinction suggests that the functional engagement of this circuit depends critically on the presence of psychological stress. To interpret this finding, we referred to the clinical distinction between peripherally initiated IBS and centrally initiated IBS [5, 51, 52, 53, 54, 55]. Peripherally initiated IBS, including diet‐related and post‐infectious IBS, involves local immune activation and represents a “gut‐to‐brain” pathway where peripheral immune responses drive symptom onset in the absence of significant psychological distress [52, 53, 56]. The OVA‐induced model, which exhibits visceral pain without anxiety‐like behavior, thereby representing this clinical subtype. In this context, the ACC may function as a general sensory hub for processing visceral afferent signals, working together with other downstream pathways, such as the ACC‐periaqueductal gray (PAG)‐rostral ventromedial medulla (RVM)‐spinal cord descending circuit, without necessarily involving the ACC‐AIC projection [13, 18, 57]. This explains why ACC inhibition is effective in both models, whereas the stress‐specialized ACC^Glu^‐AIC^Glu^ circuit is not prominently involved in the OVA model. In contrast, centrally initiated IBS represents a “brain‐to‐gut” pathway where psychological stress initiates or exacerbates symptoms through central nervous system mechanisms [6, 51, 54, 55]. The WAS model, which induces both visceral hypersensitivity and anxiety‐like behavior, represents this clinical subtype. Notably, the ACC^Glu^‐AIC^Glu^ circuit is not activated by pain signals alone but requires an additional affective or stress component, which aligns with the established role of the AIC in emotional processing and affective‐sensory integration [50, 58]. Under chronic psychological stress, the ACC‐AIC circuit becomes hyperactive, integrating sensory and affective information and thereby amplifying visceral pain via descending facilitation. Consequently, inhibition of this circuit alleviates both visceral hypersensitivity and anxiety‐like behavior. Taken together, the ACC serves as a common hub for visceral pain, while the ACC^Glu^‐AIC^Glu^ circuit is a stress‐specific amplifier for pain‐emotion integration rather than an essential relay for visceral pain perception.

The trafficking of iGluRs, particularly AMPARs and NMDARs, is dynamically regulated by synaptic activity, thereby modulating synaptic strength and plasticity [59, 60]. Our study demonstrated that WAS enhanced the synaptic expression of GluA1/GluA3‐containing AMPARs and NR2B‐containing NMDARs in the AIC. Unlike the Ca^2+^ impermeable subunit of GluA2, GluA1/A3 subunits are Ca^2^^+^‐permeable. Therefore, calcium‐permeable AMPARs (CP‐AMPARs), including GluA1 homomers or GluA1/A3 heteromers lacking GluA2 subunits, exhibit high conductance and activate Ca^2+^‐dependent signaling pathways [61]. The membrane insertion of CP‐AMPARs and NR2B‐containing NMDARs in multiple brain regions has been shown to mediate long‐term potentiation underlying chronic pain and emotional disorders [48, 62, 63, 64, 65]. Furthermore, previous studies have demonstrated that inhibition of glutamatergic transmission by injecting NMDA or AMPA antagonists into the AIC has analgesic effects [48, 66]. In the present study, we found that activation of the ACC‐AIC glutamatergic pathway promoted CP‐AMPARs and NR2B trafficking to the synaptic membrane of AIC neurons and enhanced the postsynaptic NMDAR and AMPAR currents, whereas block of this pathway reversed these effects. Additionally, inhibition of CP‐AMPARs and NR2B by microinjecting NASPM and Ro25‐6981 into AIC successfully mitigated the visceral hypersensitivity and anxiety caused by WAS. These observations suggest that activation of the ACC‐AIC glutamatergic pathway may enhance calcium influx in post‐synaptic neurons through facilitating synaptic incorporation of GluA1‐ and GluA3‐containing CP‐AMPARs and NR2B‐containing NMDARs at AIC synapses, which leads to potentiation of AIC synaptic transmission, thus resulting in visceral hypersensitivity and anxiety.

The present study investigated the fundamental role of the ACC^Glu^‐AIC^Glu^ circuit in the comorbidity of visceral pain and anxiety. However, some limitations should be acknowledged. One limitation concerns the pharmacological agents employed. Although NASPM and Ro25‐6981 were applied to target synaptic CP‐AMPARs and NR2B, their potential effects on extra‐synaptic receptors cannot be completely ruled out. The absence of direct interventions for modulating the synaptic expression of CP‐AMPARs and NR2B limits the definitive conclusions regarding the synaptic mechanism. Additionally, while our tracing studies confirmed reciprocal projections between the ACC and AIC, the functional significance of the weaker AIC to ACC projection remains to be elucidated. This pathway may be involved in reciprocal modulation or other aspects of interoceptive processing, representing an important direction for future investigation.

Furthermore, the contribution of GABAergic neurons in the ACC and AIC in modulating visceral pain and anxiety merits further investigation to help develop a comprehensive map of the neural substrates underlying the comorbidity of visceral hypersensitivity and anxiety in IBS patients.

## 4 Conclusion

Our study concluded that hyperactivity in ACC and AIC glutamatergic neurons, especially within the ACC‐AIC glutamatergic circuit, was crucial for mental stress‐induced visceral hypersensitivity and anxiety. Notably, this circuit was not implicated in OVA‐induced visceral hypersensitivity, a model in the absence of anxiety, showing its specific role in psychophysiological comorbidity. More importantly, we found a novel mechanism in which the trafficking of GluA1/GluA3‐containing CP‐AMPARs and NR2B‐containing NMDARs at AIC synapses mediated this circuit's dysfunction. These findings uncover a link between synaptic plasticity and circuit‐level dysfunction, thus establishing a “circuit‐to‐synapse” framework that facilitates the understanding of sensory‐emotional integration. Additionally, glutamate receptor plasticity in the ACC‐AIC sensory‐emotional gating circuit may have implications in the treatment of comorbid pain and anxiety disorders, as well as other conditions with sensory–affective interactions.

## 5 Materials and Methods

### 5.1 Experimental Animals

Male Wistar rats (SLAC Laboratory Animal Co., Ltd, Shanghai, China) with body weight 120–200g were housed under specific pathogen‐free (SPF) conditions with a constant temperature (24±2 °C) and relative humidity, subjected to a reversed 12h light/dark cycle with lights on at 7:00 am. The animals had ad libitum access to food and water except for a fasting period before visceral sensation assessment. All experimental protocols were conducted according to the guidelines approved by the Animal Care and Use Committee of Tongji University (Shanghai, China,Approval number: TJBC00823201).

### 5.2 Model Establishment

Visceral hypersensitivity was induced by chronic water avoidance stress (WAS) in rats, as previously described [11, 15]. In brief, a polygonal‐shaped platform (8 cm length × 8 cm width × 10 cm height) was fixed in the center of each Plexiglas water tank (45 cm length × 25 cm width × 25 cm height). The tanks were filled with fresh water up to 1 cm below the tops of the platforms. The WAS rats were placed on the platforms for an hour (between 9:00 and 12:00 am) every day, for 10 consecutive days. The SHAM rats were subjected to an identical procedure but without water in the tanks. Rats in the normal control (NC) group were left undisturbed (Figure 1A).

The Ovalbumin (OVA) model is a well‐established model for studying non‐mental stress‐induced visceral pain [44]. Briefly, rats were sensitized to OVA by intraperitoneal injection of a solution containing OVA (100µg, MCE, Monmouth Junction, NJ, USA) as the antigen and aluminum hydroxide (10mg, MCE, Monmouth Junction, NJ, USA) as an adjuvant in 1mL saline. Beginning on day 3 after sensitization, a daily intracolonic administration of 100 µg OVA in 1 mL saline was performed for three consecutive days using a medical catheter with an outer diameter of 3 mm. To prevent the outflow of OVA, the rats were maintained in an inverted position for 3 min. Control rats were subjected to an identical procedure but without antigen or adjuvant contained in the saline solution. The rats were allowed a recovery period for 6 days before the behavior tests.

### 5.3 Behavioral Tests

Rats were moved to the behavioral laboratory a day before the behavior tests to adapt to the environment. The testing equipment was cleaned with 75% ethanol and dried with a sterile cloth after each test. The movement of the rats in all tests was recorded by a video camera and subsequently analyzed using the EthoVisionXT8.0 Tracking System (Noldus Information Technology, Wageningen, the Netherlands).

#### 5.3.1 Open‐Field Test (OFT)

The open‐field test was conducted using a black‐painted wooden rectangular box (80 cm length × 80 cm width × 40 cm height) to measure locomotion and anxiety‐like behavior. The bottom surface of the arena was divided into a central zone (CZ) (40 cm length × 40 cm width) and a peripheral zone. After being gently placed in the center of the arena, each rat was allowed a 5‐min exploration period. The total movement distance, the number of entries into the CZ, and the time spent within the CZ were recorded.

#### 5.3.2 Elevated Plus Maze (EPM) test

The EPM tests were conducted using a plastic cruciform apparatus elevated 50 cm above the ground. The apparatus consisted of a central platform (10 cm length × 10 cm width), two open arms (OA, 50 cm length× 10 cm width), and two closed arms (50 cm length × 10 cm width × 40 cm height). After 5 min of acclimation in the open field, the rats were introduced to the central platform, facing one of the open arms, and their behavior was monitored for 5 min by a digital camera. The time spent in open or closed arms, and the number of entries into each type of arms were recorded. In addition, the anxiety index was calculated to assess anxiety levels, using the formula: Anxiety index = 1 – [(time spent in open arms/total time spent in the maze + number of entries to the open arms/total entries in the maze)/2].

#### 5.3.3 Forced Swim Test (FST)

The FST was used to assess depression‐like behavior in rats. To minimize interference, the FST was performed on a separate day from the OFT and EPM. During the test, rats were individually placed into a transparent cylinder filled with water (30 cm diameter × 50 cm height, 30 cm water height, and 23 ± 1°C water temperature). The movements of rats were monitored for 6 min using a digital camera. Immobility, characterized as the cessation of all movements except those essential for the rat to keep its head afloat, was used as an indicator of depression‐like behavior. The immobility time during the last 4 min was tested. The water in the cylinder was replaced after each trial to prevent any potential carry‐over effect on subsequent tests.

### 5.4 Visceral Motor Response (VMR) Assessment

Electromyograms (EMG) of external abdominal oblique muscles were recorded to indicate the VMR to colorectal distention (CRD), as described in previous studies [15, 67]. Briefly, rats were anesthetized with 2% pentobarbital sodium (50 mg/kg) right after the completion of behavior tests. A pair of Teflon‐coated silver wires were then implanted with one end in the external abdominal oblique muscle and the other end threaded through a subcutaneous tunnel and exposed to the back of the rat's neck. After suturing, the rats were housed individually to recover for 3 days. Additionally, the rats were fasting overnight the day before the EMG tests. On the test day, the rats were separately introduced into a well‐sized oval restraining device (Yuyan Instruments, Shanghai, China) and then a pre‐made latex balloon tied around a medical catheter (3mm diameter) was inserted 4–5cm into the distal colon. After 30 min of acclimatization, the VMRs to 40mmHg (an innocuous stimulus) and 60mmHg (a painful stimulus) CRD pressures were tested 3 times separately. Each test consisted of a 20‐s baseline EMG recording, a 20‐s EMG recording at a specific CRD pressure, a 20‐s post‐stimulation EMG recording, and then a 5‐min recovery period. An amplifier (Brownlee Precision Model 440, SanJose, CA, USA) and an Axon Digidata 1440A data acquisition system (Molecular Devices, Sunnyvale, CA, USA) were used to capture the EMG signal. The signal was amplified by a factor of 5000, band‐pass filtered between 50 and 500 Hz, and sampled at 1 kHz. To quantify the VMR, the difference between the area under the curve (AUC) of the baseline and the CRD period was calculated using Clamp Fit 10.7 (Molecular Devices, Sunnyvale, CA, USA).

### 5.5 Immunohistochemistry

After deep anesthesia with pentobarbital sodium (100 mg/kg), the rats were rapidly perfused by 250mL of 0.9% ice‐cold saline, followed by 4% paraformaldehyde (PFA). The brains were immediately dissected, immersed in 4% PFA overnight, and then cryoprotected in 30% sucrose at 4°C for 3 days. Coronal slices (10µm thickness) of the ACC and AIC were obtained using a cryostat microtome (Lecia, Wetzlar, Germany). The sections were washed three times in 0.01 M phosphate‐buffered saline (PBS, pH 7.4) for 5 min each, permeabilizated with 0.2% Triton X‐100 for 15 min, and blocked by QuickBlock Blocking Buffer (P0260, Beyotime, Shanghai, China) for 30 min at room temperature. Next, the slices were incubated overnight at 4°C with appropriate primary antibodies, including c‐fos (1:100, sc‐166940, Santa Cruz Biotechnology, Delaware, TX, USA), CAMKIIα (1:250, ab52476, Abcam, Cambridge, UK), or GAD65+67 (1:1000, ab239372, Abcam, Cambridge, UK). After being washed three times in PBS for 15 min each, the slices were incubated with the appropriate secondary antibodies, including Alexa Fluor 488‐labeled goat anti‐rabbit IgG (1:200, A0423, Beyotime, Shanghai, China) and Cy3‐labeled goat anti‐mouse/rabbit IgG (1:200, A0521/A0516, Beyotime, Shanghai, China) for 2 h at room temperature. Finally, the washed slices were mounted on glass slides using Antifade Mounting Medium with 4',6‐diamidino‐2‐phenylindole (DAPI) (P0131, Beyotime, Shanghai, China) and observed under a fluorescence microscope (LSM900, Zeiss, Oberkochen, Germany). For tissues infected by fluorescent viruses, all procedures were conducted under conditions that protected from light.

### 5.6 Cell‐Counting

4–6 rats for each group were used to quantify the expression of c‐fos, EGFP‐labelled virus, and co‐localization of EGFP‐labelled virus/c‐fos with CAMKIIα/GAD65+67 in neurons in the ACC or AIC. For each rat, positively stained cells in target regions were counted and averaged in three sections separated by 200µm to prevent repeat counting. Counting was performed by an investigator blinded to treatment conditions using Image J software.

### 5.7 In Vivo Viral Injections

Rats weighing about 120g (about 5 weeks old) were anesthetized with 2% pentobarbital sodium (50 mg/kg). Incisions were made in the skull upon the target sites using a dental drill to insert a micro‐syringe (0.5 ml, Hamilton, Reno, NV, USA) controlled by a syringe pump (Harvard Apparatus, Holliston, MA, USA). The virus (BrainVTA, Wuhan, China) was then microinjected into the target site at a rate of 0.08ul/min with 1µl per site. According to the brain atlas published by Paxinos and Watson [68], the coordinates were as follows: 2 mm anterior to the bregma, 0.6 mm lateral to the midline, and 2 mm ventral to the skull surface for the ACC; and 2 mm anterior to the bregma, 4.2 mm lateral to the midline, and 5.8 mm ventral to the skull surface for the AIC. After injection, micro‐syringe was left in place for 2 min to minimize back‐flow. Virus‐infected rats were allowed to recover for 5 weeks before testing. Detailed injection information is provided below.

For retrograde tracing, rAAV2/Retro‐CAG‐EGFP‐WPRE‐hGH·pA (1.21 × 10^13^vg/mL) was microinjected into unilateral ACC or AIC. In addition, for anterograde trans‐monosynaptic tracing, rAAV2/1‐hSyn‐Cre‐WPRE‐hGH·pA(1.09 × 10^13^vg/mL) was injected into the unilateral ACC and rAAV2/9‐hSyn‐DIO‐EGFP‐WPRE‐hGH·pA (5.14 × 10^12^vg/mL)was injected into the ipsilateral AIC.

For chemogenetic manipulation of local ACC or AIC glutamatergic neurons, rAAV2/9‐CAMKIIα‐hM3Dq‐EGFP‐WPRE‐hGH·pA(5.03 × 10^12^vg/mL) was injected into bilateral ACC or AIC of SHAM rats, rAAV2/9‐CAMKIIα‐hM4Di‐EGFP‐WPRE‐hGH·pA(5.09 × 10^12^vg/mL) virus was injected into bilateral ACC or AIC of WAS rats. Besides, AAV2/9‐CAMKIIα‐EGFP‐WPRE‐hGH·pA(5.07 × 10^12^vg/mL) was injected into bilateral ACC or AIC of both SHAM and WAS rats as a control.

For chemogenetic manipulation of the circuit of ACC glutamatergic neurons projecting to AIC, rAAV2/9‐CAMKIIα‐hM3Dq‐DIO‐EGFP‐WPRE‐hGH·pA(5.16 × 10^12^vg/mL) or rAAV2/9‐CAMKIIα‐hM4Di‐DIO‐EGFP‐WPRE‐hGH·pA(5.07 × 10^12^vg/mL) was injected into the bilateral ACC of SHAM or WAS rats, respectively. Furthermore, rAAV2/9‐CAMKIIα‐DIO‐EGFP‐hGH·pA(5.11 × 10^12^vg/mL) was injected into bilateral ACC of both SHAM and WAS rats as a control. Simultaneously, rAAV2/Retro‐CMV‐Cre‐EGFP‐hGH·pA(5.02 × 10^12^vg/mL) was injected into bilateral AIC of all the above rats.

For optogenetic manipulation, a combination of rAAV2/9‐ CAMKIIα‐CHR2‐mCherry‐WPRE‐hGH·pA(5.25 × 10^12^vg/mL) and rAAV2/1‐hSyn‐Cre‐WPRE‐hGH·pA (1.09 × 10^13^vg/mL) was injected into unilateral ACC, and rAAV2/9‐hSyn‐DIO‐EGFP‐WPRE‐hGH·pA(5.14 × 10^12^vg/mL) was injected into ipsilateral AIC.

Only rats displaying good virus expression were included for further analyses.

### 5.8 In Vitro Slice Electrophysiological Recordings

The procedures for slice electrophysiological recordings were based on a previous study [69]. Briefly, rats were anesthetized with 2% isoflurane and decapitated. Coronal brain slices of ACC and AIC (300µm thickness) were obtained using a vibratome (VT1000S, Leica, Wetzlar, Germany) in a cold cutting solution containing (in mM): sucrose 220, KCl 2.5, NaH_2_PO_4_ 1.25, CaCl_2_ 0.5, MgCl_2_ 4, NaHCO_3_ 26, and glucose 10. The slices were transferred to artificial cerebral spinal fluid (ACSF) saturated with 95% O2, and 5% CO2, which contained (in mM): NaCl 119, KCl 2.5, NaH_2_PO_4_ 1.25, CaCl_2_ 2.5, MgCl_2_ 1.3, NaHCO_3_ 26, and glucose 10 at room temperature. After a minimum incubation time of 1 h, whole‐cell recordings were performed with recording pipettes (3‐5 MΩ) filled with an internal solution containing (in mM): K‐gluconate 105, KCl 30, HEPES 10, EGTA 0.3, phosphocreatine‐Na_2_ 10, ATP‐Mg 4, GTP‐Na 0.3 (adjusted to pH 7.35 with KOH, 285 mOsm/kg). The EGFP‐ or mCherry‐labeled neurons could be visualized using a water‐immersion objective (×40) on an upright microscope (BX51WI, Olympus, Tokyo, Japan). Depolarizing current pulses (400 ms duration) from 0 to 200 pA were applied in increments of 50 pA to study the stimulus‐firing relationship of AIC‐projecting ACC glutamatergic neurons in WAS and SHAM rats. The threshold of stimulation was defined as the minimum current required to evoke action potentials. Light‐evoked firing and light‐evoked EPSCs were recorded in ACC glutamatergic neurons and AIC neurons which received ACC fibers, respectively.

To record puff‐evoked AMPAR and NMDAR currents, lidocaine N‐ethyl bromide QX‐314(5mM, MCE, Monmouth Junction, NJ, USA) was added to the internal solution to block action potentials. The tips of the puff micropipettes (∼2µm) were positioned 100–150µm away from the recording cells. AMPA (100µM, MCE, Monmouth Junction, NJ, USA) or NMDA (500µM, MCE, Monmouth Junction, NJ, USA) was ejected (2psi, durations of 1s) through a positive pressure system (Picospritzer, Parker Hannifin, Hollis, NH, USA) onto the selected neuron. Evoked AMPAR currents were recorded at a holding potential of −70 mV in the presence of 10µM GABA_A_ receptor antagonist picrotoxin (Sigma, USA) and 50µM NMDAR antagonist D‐2‐Amino‐5‐phosphonovaleric acid (D‐AP5, MCE, Monmouth Junction, NJ, USA). Evoked NMDAR currents were recorded at a holding potential of −30 mV in the presence of 10µM picrotoxin and 10µM AMPAR antagonist 6,7‐dinitroquinoxaline‐2,3‐dione (DNQX, MCE, Monmouth Junction, NJ, USA). The puff‐evoked AMPAR or NMDA currents of cells from rats expressing the DREADD virus were recorded before or 10 min after CNO perfusion.

The recordings were conducted using an Axopatch‐700B amplifier (Molecular Devices, Sunnyvale, CA, USA) with a sampling rate of 10 kHz and filtered at 2 kHz. Clampfit 10.7 software (Molecular Devices, Sunnyvale, CA, USA) were used for data analysis.

### 5.9 Chemogenetic and Optogenetic Neuronal Manipulation

For the chemogenetic neuronal manipulation, rats expressing a chemogenetic or control virus received an intraperitoneal injection of 2 mg/kg Clozapine N‐oxide (CNO, C0832, Sigma–Aldrich St. Louis, MO, USA) 45 min before behavioral or VMR tests. To compare the VMRs of the same rat before and after intervention, the baseline VMRs were pre‐recorded a day before CNO injections.

Optical stimulation (470 nm, 15ms) of the patched cell was applied through the X‐Cite 120 LED system (Lumen Dynamics, Mississauga, Canada) attached to the microscope using a 3‐Hz stimulation protocol with a pulse width of 15ms. The opto‐stimulation induced excitatory postsynaptic currents (oEPSCs) were recorded before and after perfusion with DNQX (10Μm, Monmouth Junction, NJ, MCE), to confirm the oEPSCs were AMPA currents.

### 5.10 Quantitative Reverse Transcription Polymerase Chain Reaction (qRT‐PCR)

After VMR assessments, the rats were sacrificed within 30 min. The AIC tissues were quickly dissected under RNase‐free conditions. The PrimeScript^TM^ RT Master Mix (RR036, TakaRa, Kusatsu, Japan) was used to reverse‐transcribe the mRNA extracted with Trizol and capture the corresponding cDNA. The PCR reactions were carried out with TB Green^TM^ Premix ExTaq^TM^ (RR420, TaKaRa, Kusatsu, Japan) via QuantStudio7 Flex RealTime PCR System (Applied Biosystems, Waltham, MA, USA). The 2^−ΔΔCT^ method was used for data analysis with GAPDH as the internal reference. The primer sequences used were as follows:

NR1 (F: TGGTGGCAGATGGCAAGTTTGG, R: ACGCTCATTGTTGATGGTCAGTGG)
NR2A (F: TCCAGCAGCAAGCCACAGTTATG, R: TGAAGTCTCGGTAGCCAGGGAAG)
NR2B (F: AGGAGGAAGTAAGACCAGCACAGG, R: AGGAAGCGGGAGGCAAATGAATG)
GluA1 (F: AGTCCAAGCCAGGTGTCTTCTCC, R: CTCTTCGCTGTGCCATTCGTAGG)
GluA2 (F: GCATTTCGGGTAGGGATGGTTCAG, R: TGGGAGCAGAAAGCATTGGTGAC)
GluA3 (F: CGCTGGGTGAGACTGGATGAAAG, R: GGTATCGGAAGGCTTCTGCTATGAC)

### 5.11 Western Blot

To obtain total proteins, the samples harvested from the AIC were lysed by cold lysis buffer (radioimmunoprecipitation assay: phenylmethylsulfonyl fluoride = 100:1) on ice.

To extract synaptosomal fraction, we used the Syn‐PER Reagent (87793, Thermo Scientific, Waltham, MA, USA) with protease and phosphatase inhibitors (50×) as the lysis buffer. The samples were centrifuged at 1200 G for 10 min, and the resulting supernatant was then centrifuged at 15 000 G for 20 min at 4°C to obtain the synaptosome pellet. Finally, the pellet was resuspended in 100µL of the lysis buffer.

The concentration of all samples was quantified by bicinchoninic acid (BCA) Protein Assay Kit (P0010S, Beyotime, Shanghai, China). Then, the proteins were heated at 100°C for 15 min in sodium dodecyl sulfate polyacrylamide gel electrophoresis (SDS‐PAGE) Sample Loading Buffer (P0286, Beyotime, Shanghai, China) and subjected to SDS‐PAGE for 30 min at 220 V. Proteins were transferred to a polyvinylidene difluoride (PVDF) membrane for 30 min at 400pA. Membranes were blocked with 5% non‐fat milk in Tris‐buffered saline with Tween 20 (TBST) for 1 h at room temperature and then incubated overnight at 4°C with the following primary antibodies: anti‐NR1 (1:1000, ab109182, Abcam, UK), anti‐NR2A (1:1000, ab124913, Abcam, Cambridge, UK), anti‐NR2B (1:1000, ab65783, Abcam, Cambridge, UK), anti‐GluA1 (1:10000, 67642‐1‐Ig, Proteintech, Wuhan, China), anti‐GluA2 (1:2000, ab133477, Abcam, Cambridge, UK), anti‐GluA3 (1:1000, 29588‐1‐AP, Proteintech, Wuhan, China), PSD95 (1:1, 000, ab36233, Cambridge, Abcam), PCNA(1:1000 10205‐2‐AP, Proteintech, Wuhan, China) or anti‐β‐Tubulin (1:1000, ARG62347, Arigo Biolaboratories Co., China). Following primary antibody incubation, membranes were probed with horseradish peroxidase (HRP)‐conjugated goat anti‐mouse/rabbit IgG antibody (1:5000, ARG65350/65351, Arigo Biolaboratories Co., Shanghai, China). Gel images were visualized through an enhanced chemiluminescence (ECL) Kit (WBULS0100, Millipore, Billerica, MA, USA) and analyzed using the ImageJ software.

### 5.12 Cannulation and intra‐AIC microinjection

Rats were implanted with cannulas following the established methodology [15]. Under anesthesia induced by 2% pentobarbital sodium (50 mg/kg), the rats were placed in a stereotaxic apparatus for bilateral AIC targeting based on the standard brain atlas (2mm anterior to bregma, ±4.2 mm lateral to midline, and 5.7 mm ventral from the skull surface). Two 7.5 mm stainless‐steel guide cannula (27‐gauge, RWD Life Science Co., Ltd, Shenzhen, China) were implanted into bilateral AIC and affixed with dental cement anchored by four skull screws. After surgery, the rats were allowed to rest for 7 days to recover. At the end of the experiments, the brains were sectioned to verify the cannula locations. Data with injection sites outside of the AIC were excluded from analysis.

Microinjections were performed via two injection cannulas extending 0.5 mm beyond the tip of the guide cannulas under mild anesthesia (2% isoflurane). A mixture of 1‐naphthylacetyl spermine (NASPM, 10mM, MCE, Monmouth Junction, NJ, USA) [34] and Ro25‐6981(0.6mM, MCE, Monmouth Junction, NJ, USA) [35] was microinjected into the AIC at a rate of 0.08 uL/min with a total volume of 1µL per side. The reagents were dissolved in a 10% dimethyl sulfoxide (DMSO) solution diluted with PBS, which also served as the solvent control in this study. After microinjections, the injection cannulas were left in place for 3 min to minimize backflow.

### 5.13 Statistical Analysis

The data were presented as mean ± standard error of the mean (SEM). Normality was assessed using the Shapiro–Wilk test, and homogeneity of variances was evaluated using Levene's test. For comparisons between two groups, data with normal distribution were analyzed using either the two‐tailed unpaired or paired Student's *t*‐test, while non‐normally distributed data were analyzed using the Mann–Whitney U test or the Wilcoxon matched‐pairs signed rank test. For multiple‐group comparisons, one‐way ANOVA followed by Tukey's post‐hoc test was used for normally distributed data, whereas the Kruskal–Wallis test followed by Dunn's post‐hoc test was employed for non‐normally distributed data. The GraphPad Prism 9 program was used for all statistical analysis, and *p*‐values of less than 0.05 were regarded as significant. The sample sizes and statistical details were presented in the figure legends.

## Author Contributions

J.W., G.D., and H.S. shared co‐first authorship. S.X., and Y.H. are co‐senior authors and contributed equally to this work. Conceptualization: J.W., H.S., and S.X. Methodology: J.W., G.D., Z.D., X.L., and Y.H. Investigation: J.W., G.D., H.S., and Q.W. Visualization: J.W., and G.D. Supervision: H.S., Y.C., Y.H., and S.X. Writing (original draft): J.W., G.D., and Y.H. Writing (review & editing): J.W., G.D., H.S., Y.H and S.X.

## Funding

This work was supported by grants from Clinical Research Plan of SHDC (grant No. SHDC2022CRT004), Science and Technology Innovation Action Plan of STCSM (grant No. 22DZ2203900, grant No.22Y11908300), the National Natural Science Foundation of China (grant No. 31972914, 81974067), Independent and Original Basic Research of Tongji University (grant No. 15082150043), The 19th Experimental Teaching Reform Project of Tongji University (grant No. 1508104012), Shanghai Science and Technology Commission Project (grant No. 21Y11908500), Shanghai Science and Technology Innovation Action Plan (Project for Popular Science, grant No. 24DZ2300200), Shanghai Municipal Commission of Economy and Informatization Project (Demonstration Project for the Application of Innovative Medical Devices, grant No. 23SHS03100, 23SHS03100‐01, 23SHS03100‐03), Shanghai Tongji Hospital Clinical “Five New” Innovation and R&D Project (grant No. ITJ(ZD)2409), China Scholarship Council (grant No. 202406260236).

## Conflicts of Interest

All other authors declare they have no conflicts of interest.

## Supporting information




**Supporting File 1**: advs76298‐sup‐0001‐SuppMat.docx.

**Supporting File 2**: advs76298‐sup‐0002‐FigureS1.tif.

**Supporting File 3**: advs76298‐sup‐0003‐FigureS2.tif.

**Supporting File 4**: advs76298‐sup‐0004‐FigureS3.tif.

**Supporting File 5**: advs76298‐sup‐0005‐FigureS4.tif.

## Data Availability

All data are available in the main text or the supplementary materials. Additional data related to this study can be obtained from the corresponding authors upon reasonable request.
