# Supplementary material for: An Anterior Cingulate Cortex‐Anterior Insular Cortex Glutamatergic Circuit Gates Stress‐Induced Visceral Hypersensitivity and Anxiety via Ionotropic Glutamate Receptors Trafficking
Source: Adv Sci (Weinh). 2026 Jul 29;13(52):e76298. doi: 10.1002/advs.76298 (PMC13418048; doi:10.1002/advs.76298)
Supplement: Supplementary file 1 [file ADVS-13-e76298-s005.docx]

Supplementary Materials for

**An Anterior Cingulate Cortex-Anterior Insular Cortex Glutamatergic Circuit Gates Stress-Induced Visceral Hypersensitivity and Anxiety via Ionotropic Glutamate Receptors Trafficking**

Junwen Wang *et al.*

*Corresponding author: Shuchang Xu, xschang@163.com; Ying Huang, yinghuang@tongji.edu.cn

**This PDF file includes:** Figure S1 to S4

Figure S1. ACC^Glu^-AIC^Glu^ pathway fails to regulate the total expressions of iGluRs in AIC. (A) mRNA levels of AMPA (NR1, NR2A and NR2B) and NMDA (GluA1, GluA2 and GluA3) receptors in AIC of WAS and SHAM rats (Mann Whitney test, GluA1, *p*=0.092; GluA2, *p*=0.001; unpaired Student’s t test, GluA3, t _(12)_ =1.472, *p*=0.1668; NR1, t _(12)_ =0.7856, *p*=0.4474; NR2A, t _(12)_ =0.5728, *p*=0.5774; NR2B, t _(12)_ =1.107, *p*=0.2899). (B-C) Total (‘T’) protein levels of AMPA and NMDA receptors in AIC of WAS and SHAM rats. Representative Western blots in B, quantification in C (unpaired Student’s t test, C, T-GluA1, t _(10)_ =1.298, *p*=0.2234; T-GluA2, t _(10)_ =3.717, *p*=0.004; T-GluA3, t _(10)_ =0.2183, *p*=0.8316; T-NR1, t _(10)_ =0.9131, *p*=0.3827; T-NR2A, t _(10)_ =0.9681, *p*=0.3558; T-NR2B, t _(10)_ =0.9704, *p*=0.3547). (D) The successful enrichment and isolation of the synaptosomal fractions. PSD95, PCNA and β-Tubulin in total(‘T’), cytosol(‘C’) and synaptic(‘S’) proteins extracted from the AIC tissue of rats were analyzed by Western blot. (E-G) Total protein levels of AMPA and NMDA receptors in AIC after chemogenetic modulation of ACC-AIC pathway. Representative Western blots in E, quantification in F and G (unpaired Student’s t test or Mann Whitney test, F, T-GluA1, t _(6.323)_ =1.471, *p*=0.1893; T-GluA2, t _(10)_ =0.1829, *p*=0.8585; T-GluA3, *p*=0.0931; T-NR1, t _(5.252)_ =0.5976, *p*=0.5749; T-NR2A, *p*=0.5887; T-NR2B, t _(10)_ =0.6707, *p*=0.5176; G, T-GluA1, t _(5.650)_ =1.564, *p*=0.172; T-GluA2, t _(10)_ =0.36, *p*=0.7264; T-GluA3, t _(10)_ =0.7460, *p*=0.4728; T-NR1, *p*=0.3939; T-NR2A, t _(6.309)_ =1.693, *p*=0.1390; T-NR2B, *p*=0.6991). Data presented as mean ± SEM. (*n*=7 rats for A and *n*=6 rats for C, F, G). ***p*<0.01, ****p*<0.001.

Figure S2. No significant difference is found in anxiety or depression-related behaviors among OVA, SALINE, and NC rats. (A-D) Results of OFT. Representative exploration traces in A, statistical results of total distance in B, frequency of entries to CZ in C, time spent in CZ in D (one-way ANOVA, B, F _(2, 17)_ = 0.4125, *p*=0.6684; C, F _(2, 17)_ = 0.2153, *p*=0.8085; Kruskal-Wallis test, D, *p*=0.3058). (E-H) Results of EPM. Representative exploration traces in E, percentage of entries to OA in F, percentage of time spent in the OA in G, statistical results of the anxiety index in H (Kruskal-Wallis test, F, *p*=0.8211; one-way ANOVA, G, F _(2, 17)_ = 1.334, *p*=0.2897; H, F _(2, 17)_ = 0.3688, *p*=0.6969). (I) Immobility time during FST (one-way ANOVA, F _(2, 17)_ = 0.1688, *p*=0.846). All data are shown as mean ± SEM (*n*=6 rats for OVA and SALINE groups, *n*=8 rats for NC).

Figure S3. Inhibition of ACC^Glu^ fails to influence the anxiety or depression-like behaviors of OVA rats. (A) Representative exploration traces in OFT and EPM. (B-D) Statistical results of OFT. Statistical results of total distance in B, frequency of entries to CZ in C, time spent in CZ in D (unpaired Student’s t test, B, t _(10)_ =0.8528, *p*=0.4137; C, t _(10)_ =1.029, *p*=0.3276; D, t _(10)_ =0.7447, *p*=0.4736). (E-G) Statistical results of EPM. Percentage of entries to OA in E, percentage of time spent in the OA in F, statistical results of the anxiety index in G (Mann Whitney test, E, *p*=0.8918; unpaired Student’s t test, F, t _(10)_ =0.9799, *p*=0.3502; G, t _(10)_ =0.8820, *p*=0.3985). (H) Immobility time during FST (unpaired Student’s t test, t _(10)_ =0.2082, *p*=0.8393). All data are shown as mean ± SEM (*n*=6 rats).

Figure S4. Inhibition of ACC^Glu^-AIC^Glu^ displays no effect on the anxiety or depression-like behaviors of OVA rats. (A) Representative exploration traces in OFT and EPM. (B-D) Statistical results of OFT. Statistical results of total distance in B, frequency of entries to CZ in C, time spent in CZ in D (unpaired Student’s t test, B, t _(10)_ =0.4988, *p*=0.6287; D, t _(10)_ =0.9296, *p*=0.3745; Mann Whitney test, C, *p*=0.7619). (E-G) Statistical results of EPM. Percentage of entries to OA in E, percentage of time spent in the OA in F, statistical results of the anxiety index in G (Mann Whitney test, E, *p*=0.2532; G, *p*=0.8182; unpaired Student’s t test, F, t _(6.368)_ =1.760, *p*=0.126). (H) Immobility time during FST (unpaired Student’s t test, t _(10)_ =0.2795, *p*=0.7856). All data are shown as mean ± SEM (*n*=6 rats).
