# Supplementary figures and images for: An Anterior Cingulate Cortex‐Anterior Insular Cortex Glutamatergic Circuit Gates Stress‐Induced Visceral Hypersensitivity and Anxiety via Ionotropic Glutamate Receptors Trafficking
Source: Adv Sci (Weinh). 2026 Jul 29:e76298. Online ahead of print. doi: 10.1002/advs.76298 (PMC13418048; doi:10.1002/advs.76298)

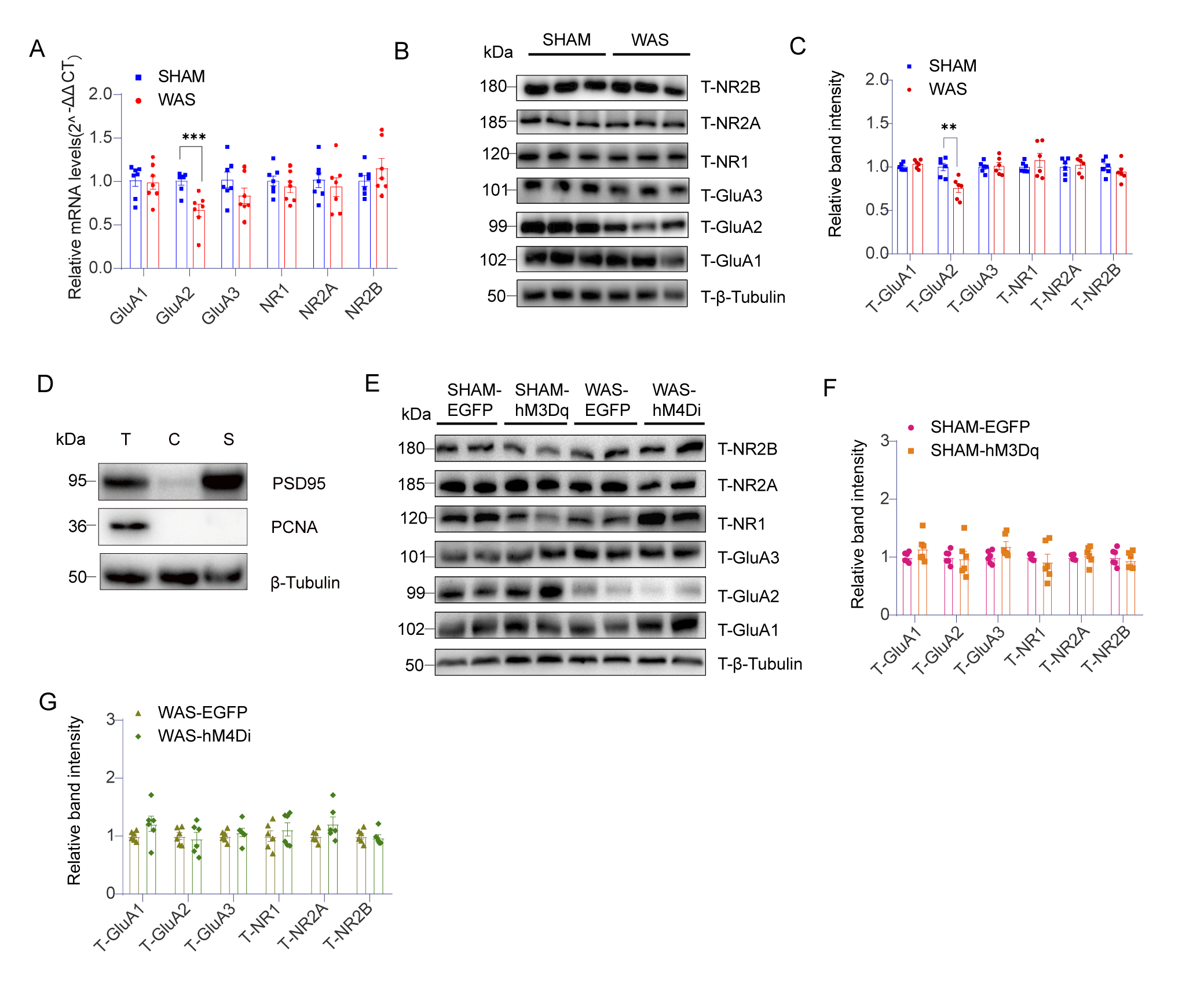

Supplement: Supplementary file 2 — Supporting File 2: advs76298‐sup‐0002‐FigureS1.tif. [file ADVS-9999-e76298-s001.tif]

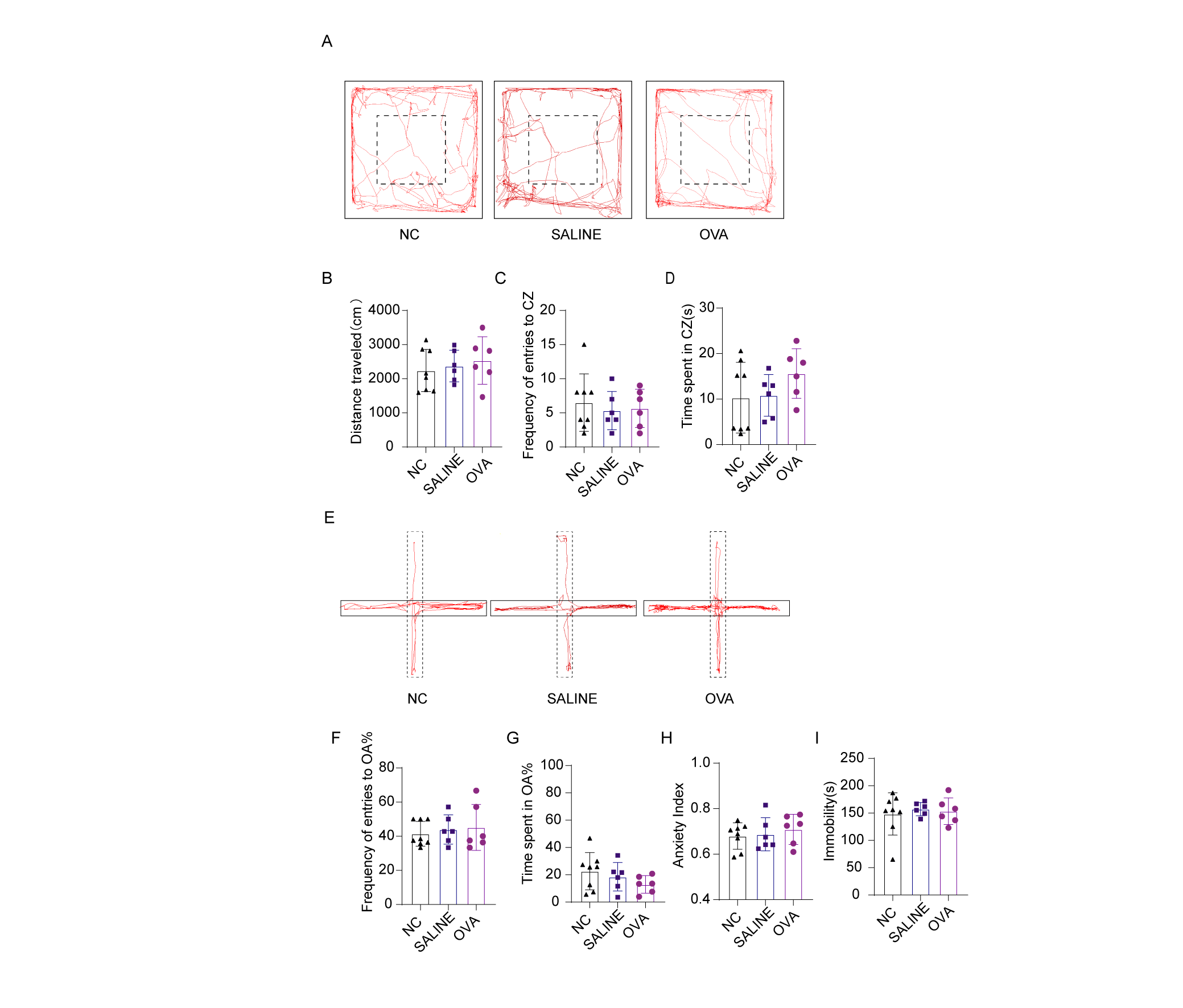

Supplement: Supplementary file 3 — Supporting File 3: advs76298‐sup‐0003‐FigureS2.tif. [file ADVS-9999-e76298-s005.tif]

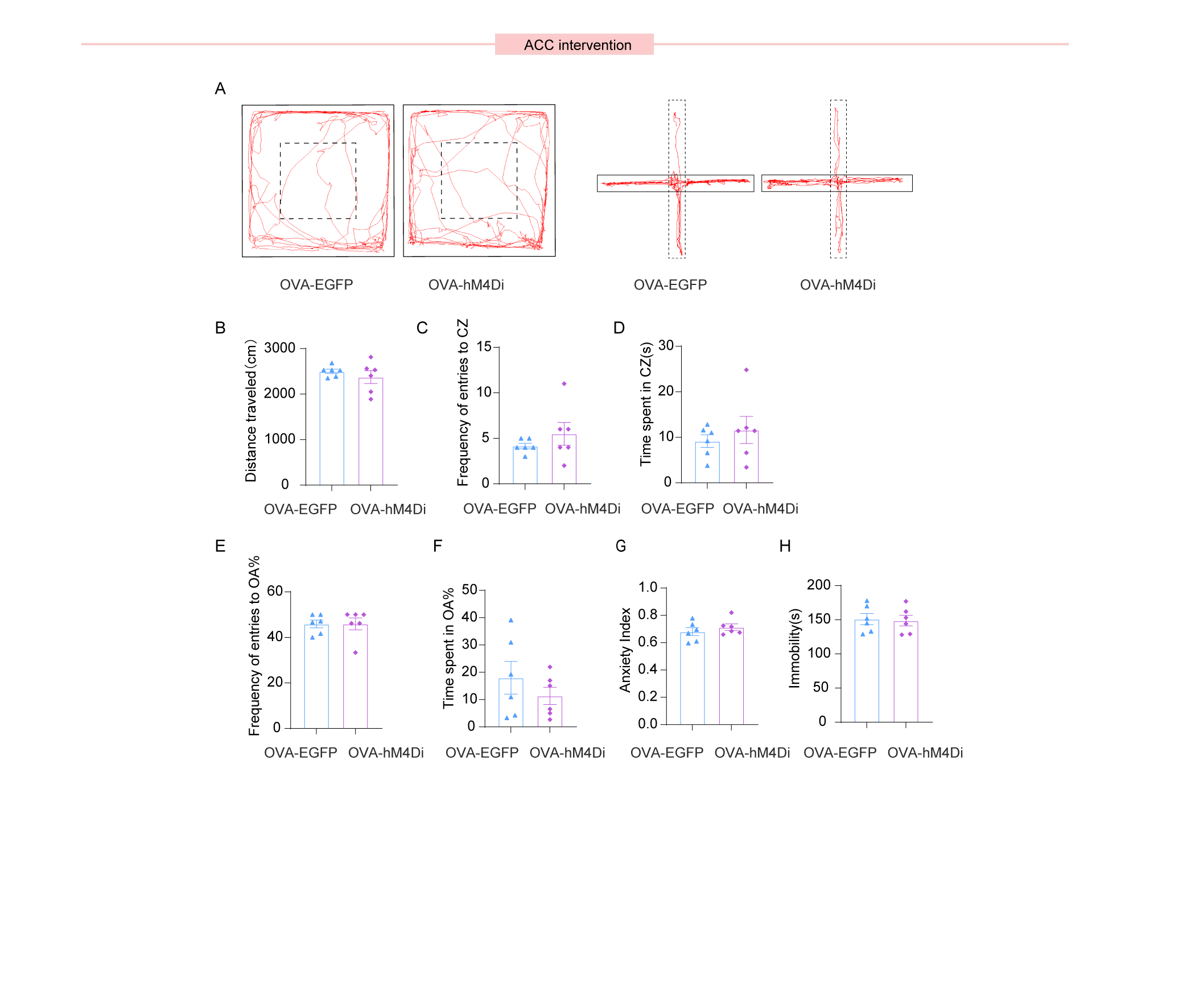

Supplement: Supplementary file 4 — Supporting File 4: advs76298‐sup‐0004‐FigureS3.tif. [file ADVS-9999-e76298-s003.tif]

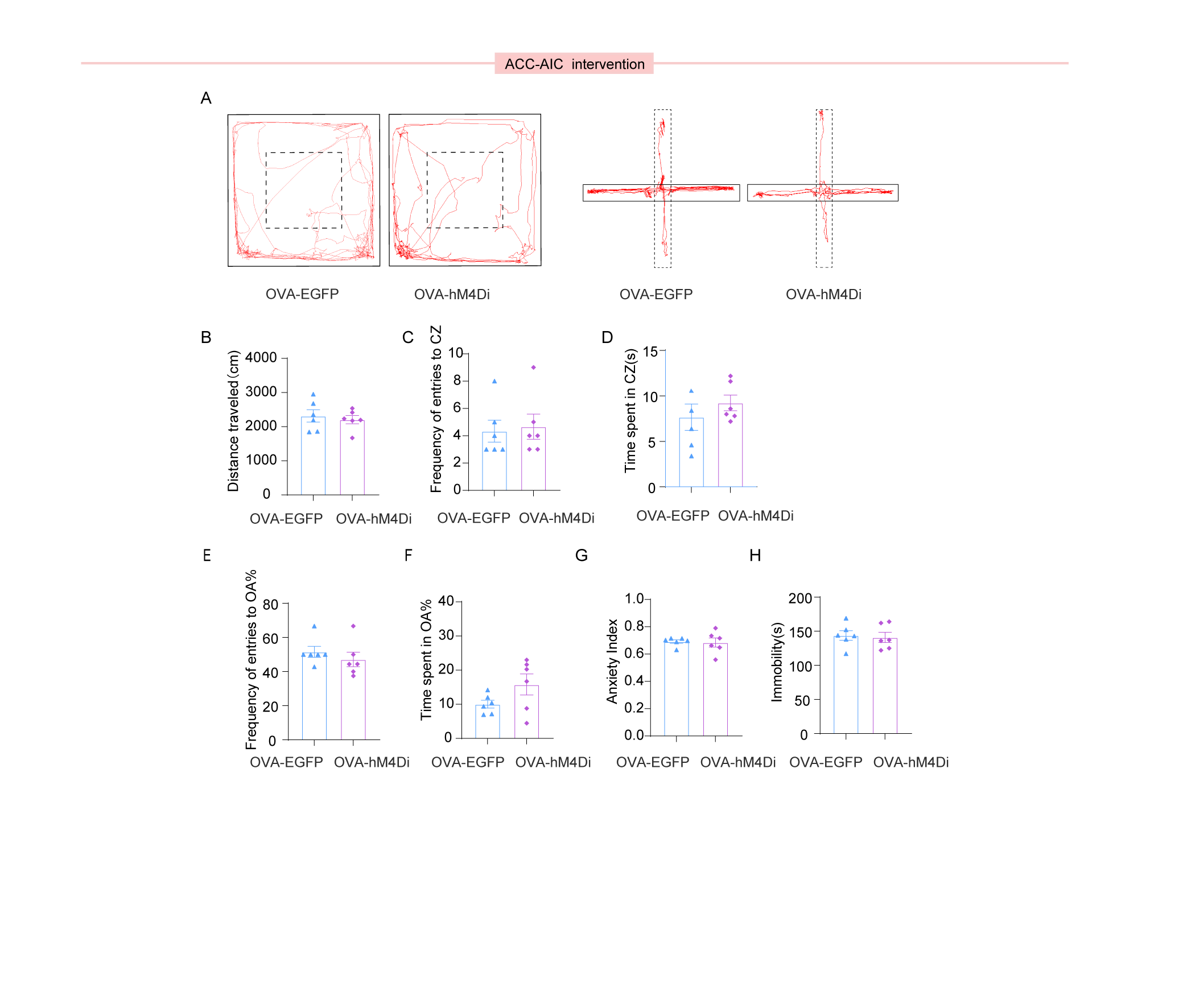

Supplement: Supplementary file 5 — Supporting File 5: advs76298‐sup‐0005‐FigureS4.tif. [file ADVS-9999-e76298-s004.tif]
